# Global, regional, and national burden of chronic kidney disease attributable to red meat consumption from 1990 to 2021

**DOI:** 10.3389/fnut.2025.1666684

**Published:** 2025-09-19

**Authors:** Jie Gu, Jiaying Zhang

**Affiliations:** ^1^Division of Geriatrics, Huashan Hospital, Fudan University, Shanghai, China; ^2^Division of Nutrition, Huashan Hospital, Fudan University, Shanghai, China; ^3^National Clinical Research Center for Aging and Medicine, Huashan Hospital, Fudan University, Shanghai, China

**Keywords:** chronic kidney disease, deaths, disability-adjusted life years, Global Burden of Disease Study, high red meat intake

## Abstract

**Objectives:**

High red meat is a crucial risk factor for chronic kidney disease (CKD). However, detailed reports on the burden of CKD due to red meat are limited. We aimed to assess the global, regional and national trends in CKD attributable to high red meat.

**Study design:**

A comprehensive analysis was performed using data from the Global Burden of Diseases (GBD) 2021.

**Methods:**

Age-standardized mortality rates (ASMR) and age-standardized DALYs rates (ASDR) were key parameters used to evaluate CKD burden. The estimated annual percentage change (EAPC) was calculated to evaluate the secular trends of ASMR/ASDR. We further explored the associations of socio-demographic index (SDI) with ASMR/ASDR.

**Results:**

From 1990 to 2021, CKD caused by high red meat led to a continuous rise in global ASMR/ASDR. Regionally, both ASMR and ASDR of CKD showed slight positive correlations with SDI, with correlation coefficients of 0.16 and 0.20, respectively. High SDI region experienced the heaviest disease burden, with the most significant increase in ASMR [EAPC 0.44 (95% CI: 0.36–0.52)], related to aging and epidemiological changes. Middle SDI region followed closely, with the highest ASDR increase [EAPC 0.44 (95% CI: 0.36–0.52)]. The USA had the highest number of CKD deaths due to high red meat, followed by China. Type 2 diabetes was the primary mediator linking increased red meat consumption to CKD.

**Conclusion:**

CKD attributable to high red meat consumption has led to a continuous rise in global burden from 1990 to 2021, with high and middle SDI regions bearing the brunt of the burden.

## 1 Introduction

Chronic kidney disease (CKD) is a major public health problem globally, with prevalence and incidence rates that have increased in the past 30 years by 40% (1). Recent reports suggested that there were 697 million cases of CKD globally and 41.5 million disability-adjusted life years (DALYs) associated with the disease, resulting in an enormous health and economic burden (2). CKD is not only associated with high mortality from cardiovascular disease, but also results in a significant number of healthy life years lost (3, 4).

Red meat refers to mammalian muscle, mainly including beef, lamb, pork, etc. Red meat is characterized by a high level of myoglobin, which gives it a red color in the raw meat state. Red meat is an important part of many cultural diets and is rich in protein, vitamins, and minerals. These nutrients are essential for growth, repair, and the maintenance of normal physiological functions in the body (5, 6). With economic growth, the global demand for red meat has surged in both developed and developing countries. The dangers of red meat have also attracted a lot of attention in recent years. Studies have shown that high intake of red meat is associated with an increased risk of cardiovascular disease and cancer (7, 8). The International Agency for Research on Cancer (IARC) classifies red meat as a probable human carcinogen (9).

There is a significant association between red meat intake and the risk of CKD. Studies have shown that consuming higher amounts of red meat increases the risk of developing CKD and end-stage renal disease (10, 11). This increased risk may be related to the high levels of iron in red meat. In addition, red meat is often accompanied by high levels of sodium and fat, which are considered to be one of the main factors contributing to the development of metabolic disorders and obesity-related diseases such as type 2 diabetes mellitus (T2DM), hypertension and cardiovascular diseases. The combination of these dietary factors may lead to impaired renal vascularization, steatosis and inflammation, thus increasing the incidence of CKD (12). The heating process of red meat produces a number of harmful chemicals such as heterocyclic aromatic amines, N-nitrosamines, polycyclic aromatic hydrocarbons and acrylamide, which may adversely affect kidney health (13). There are significant differences in red meat intake around the world, which are not only related to geographic location but are also closely linked to a number of factors, including the level of economic development, cultural habits and dietary preferences (14).

The Global Burden of Disease (GBD) study is a comprehensive research initiative that systematically quantifies health loss from hundreds of diseases, injuries, and risk factors across populations worldwide. Its primary aim is to provide a standardized, comparable measurement of health trends to inform policy and prioritize health systems planning. GBD 2021 data-base provides comprehensive epidemiological data on 371 diseases and 88 attributable risk factors across 204 countries and territories, spanning the years 1990 to 2021. The GBD framework employs a rigorous methodology that integrates vast amounts of epidemiological data using advanced statistical models, such as disease model-Bayesian meta-regression (DisMod-MR) 2.1 and Spatio-Temporal Gaussian Process Regression (ST-GPR), to produce estimates of incidence, prevalence, mortality, and disability (15). Previous GBD studies primarily focused on the various disease burdens associated with increased red meat consumption, and there was a lack of detailed reports on the disease burden of CKD related to increased red meat intake. Therefore, this study aims to utilize the GBD 2021 data to determine the Deaths and DALYs trends due to CKD attributable to red meat consumption at the global, regional, and national levels, stratified by sex, age, and socio-demographic index (SDI), providing a unique opportunity to explore the epidemiology of CKD related to increased red meat intake, and to offer evidence for policymakers.

## 2 Methods

### 2.1 Data resource

We retrieved data on annual deaths, DALYs, age-standardized mortality rate (ASMR), and age-standardized DALY rate (ASDR) of CKD attributable to high red meat intake, based on sex, age (5-year age groups of patients aged 25–94 and ≥95 years), in 204 countries and regions between 1990 and 2021 from the Global Health Data Exchange website.^1^ In the 2021 GBD Study, deaths and DALYs were key parameters used to evaluate the burden. Regional variations in age distribution were controlled through the implementation of ASMR and ASDR. In the 2021 GBD Study, 204 countries and territories are classified within a standardized hierarchical structure comprising 7 super-regions, 21 GBD regions, and 204 individual countries or territories. These classifications are determined by geographic proximity, epidemiological similarity, and mortality patterns. In our research, we adopted this GBD-defined framework for spatial categorization. To compare the CKD burden across different levels of socioeconomic development, we mainly used the SDI as the key analytical stratifier. The SDI is a composite indicator reflecting national-level development and is calculated by normalizing per capita income, average years of schooling, and the total fertility rate among those under 25. All countries were ranked and divided into five SDI quintiles: low, low-middle, middle, high-middle, and high. This method enabled us to examine how CKD trends due to high red meat intake vary across different development levels.

### 2.2 Definitions of CKD and high red meat intake

In the 2021 GBD Study, CKD was defined by an estimated glomerular filtration rate (eGFR) of less than 60 mL/min/1.73 m^2^ or a urinary albumin-to-creatinine ratio (uACR) greater than 30 mg/g, consistent with criteria commonly used in epidemiological studies. The definition of CKD employed in this study adhered to the International Statistical Classification of Diseases and Related Health Problems, 9th Revision (ICD-9) codes, including 403–404.9, 581–583.9, 585–585.9, 589–589.9, 753–753.3, and 10th Revision (ICD-10) codes encompassing D63.1, E10.2, E11.2, E12.2, E13.2, E14.2, I12–I13.9, N02–N08.8, N15.0, N18–N18.9, and Q61–Q62.8. In 2021 GBD, CKD was categorized as a Level 3 cause, with five Level 4 subtypes: Type 1 diabetes mellitus (T1DM), T2DM, glomerulonephritis, hypertension, and other and unspecified causes (2). Sources of clinical data on CKD included hospital records, emergency room records, insurance claims, surveys, and the Global Vital Registration System. After processing and standardizing the raw data, they were analyzed using three primary standardized modeling tools: DisMod-MR 2.1, Cause of Death Ensemble model (CODEm), and ST-GPR. Utilizing these tools, estimates for prevalence, incidence, remission, and excess mortality were calculated, with stratification according to age, sex, location, and year. Based on the 2021 Maternal GBD Risk Factor Study, high red meat intake was defined as intake of more than 23 grams (ranging from 18 to 27 grams) of red meat per day as an optional level (16). The mean daily consumption of red meat, specifically beef, pork, lamb, and goat, but excluding poultry, fish, eggs, and all processed meats, was calculated.

### 2.3 Statistical analyses

All data were reported as numbers with 95% uncertainty intervals (UIs) based on the 2.5th and 97.5th percentiles of the ordered 1,000 estimates. The average trends of ASMR and ASDR of high red meat intake-attributed CKD from 1990 to 2021 were calculated using an estimated annual percentage change (EAPC), which was widely accepted to reflect the trend of age-standardized rates (ASR) over a time interval. The natural logarithm of ASR is assumed to fit a linear regression model expressed as *y* = *α* + *βx* + *ϵ*, where *y* represents log10*f*() (ASR), *x* represents the year, and *ϵ* means the error term. Then EAPC was calculated as 100 × (exp (*β*) − 1). The 95% confidence intervals (CIs) of EAPC were estimated using the linear regression model (17, 18). If the lower bound of the 95% CI for the EAPC exceeded zero, it indicated an increasing trend in the ASMR and ASDR. In contrast, if the upper bound of the 95% CI for the EAPC fell below zero, it suggested a decreasing trend in the ASR. The ASR was deemed stable when the 95% CI encompassed zero. Pearson or Spearman correlation analyses were conducted to assess the nature of the relationship between the SDI and the burden of CKD attributable to high red meat intake, as measured by the ASMR and ASDR, across 21 regions. Statistical significance was defined as a two-sided *p*-value less than 0.05.

### 2.4 Data visualization

To illustrate the burden across various regions, we created a world map displaying ASDR, ASMR and EAPC. To identify key drivers, we performed a gender-specific decomposition analysis of three factors: population, aging, and epidemiological trends. All statistical analyses and data visualization were performed employing R software (version 4.4.2). In this study, software packages such as ggplot2, dplyr and map were used.

## 3 Results

### 3.1 Global burden

**Figure 1:**
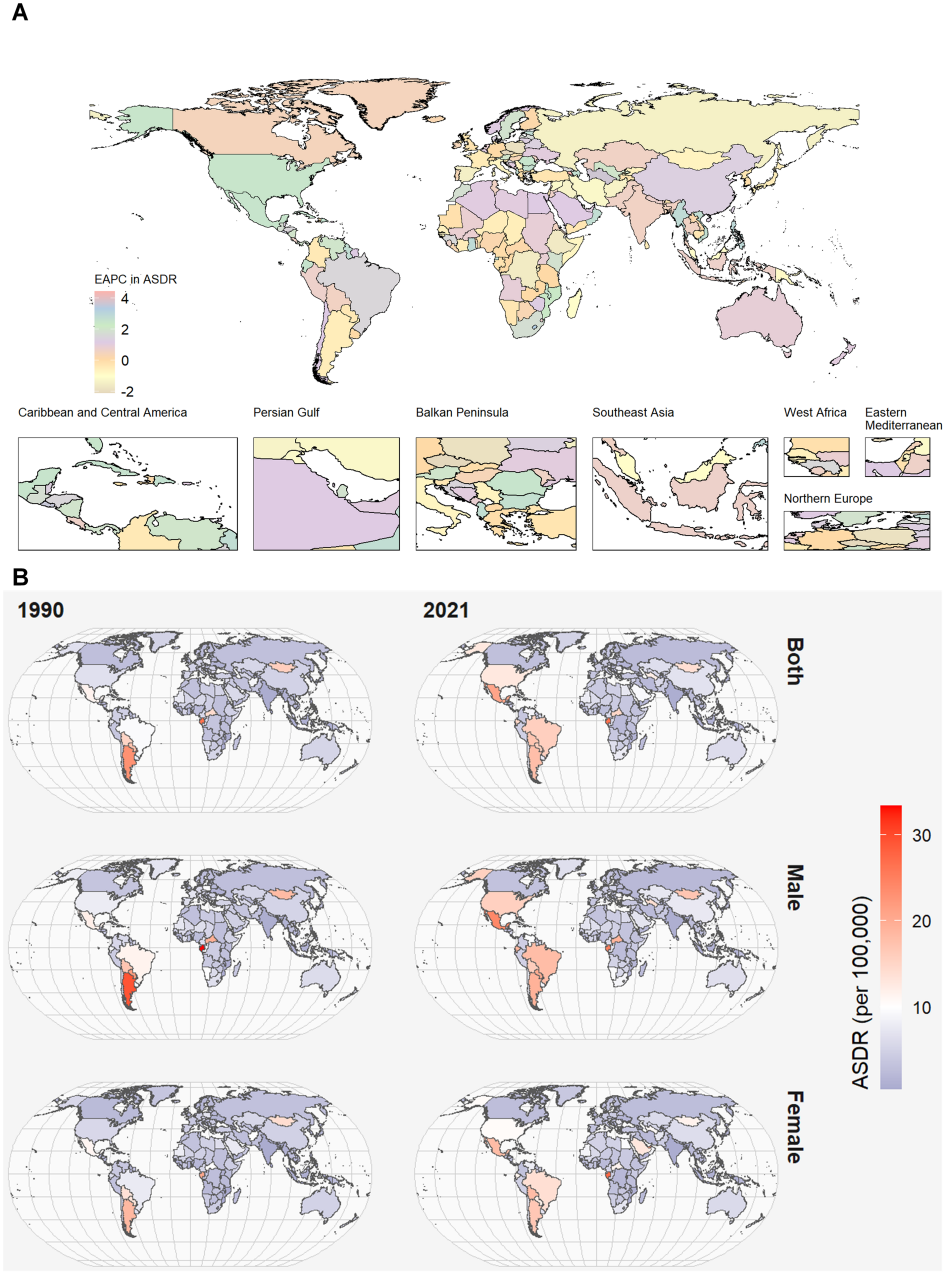
World map of ASDR of CKD attributable to high red meat intake across 204 countries and territories. **(A)** The EAPC in chronic kidney disease ASDR attributable to high red meat intake from 1990 to 2021. **(B)** The spatial distribution of chronic kidney disease ASDR attributable to high red meat intake by sex in 1990 and 2021. ASDR, age-standardized DALYs rate; DALYs, disability-adjusted life years; CKD, chronic kidney disease; EAPC, estimated annual percentage.

Compared to 1990, there has been a substantial increase in the burden of DALYs and deaths (Figure 1; Supplementary Figure 1). The deaths of CKD due to high red meat increased by 245% between 1990 and 2021. Besides, ASDR of CKD owing to high red meat in 2021 [5.5 per 100,000 (95% CI 0–12.01)] was higher than in 1990 [4.2 per 100,000 (95% CI 0–9.28)] (Supplementary Tables 1, 2).

Among 21 GBD regions, ASMR and ASDR were the highest in Southern Latin America (SLA) and Tropical Latin America (TLA). In contrast, South Asia had the lowest ASMR and ASDR. High-income North America presented the most significant annual increasing trends in ASMR and ASDR (Figure 1; Supplementary Figure 1). Moreover, the largest decline in the annual changing trends in ASMR was found in the Central Sub-Saharan Africa, with an EAPC of −0.81 (95% CI: −1 to −0.61) in ASMR. The largest decline in the annual changing trends in ASDR was found in the Eastern Europe, with an EAPC of −0.79 (95% CI, −1.04 to −0.53) in ASDR (Supplementary Tables 1, 2).

At the national level, Mexico and Northern Mariana Islands had the highest CKD burden in terms of ASDR, while Sri Lanka, Bangladesh, Tajikistan, and India had the lowest. China, followed by the United Stated of America (USA), had the greatest number of deaths of CKD attributable to high red meat both 1990 and 2021 (Supplementary Table 3).

### 3.2 Global trends by SDI

Except for the low SDI region, the DALYs burden of CKD have experienced a tremendous increase from 1990 to 2021, with the highest increase detected in the middle SDI quintile (Supplementary Table 2).

**Figure 2:**
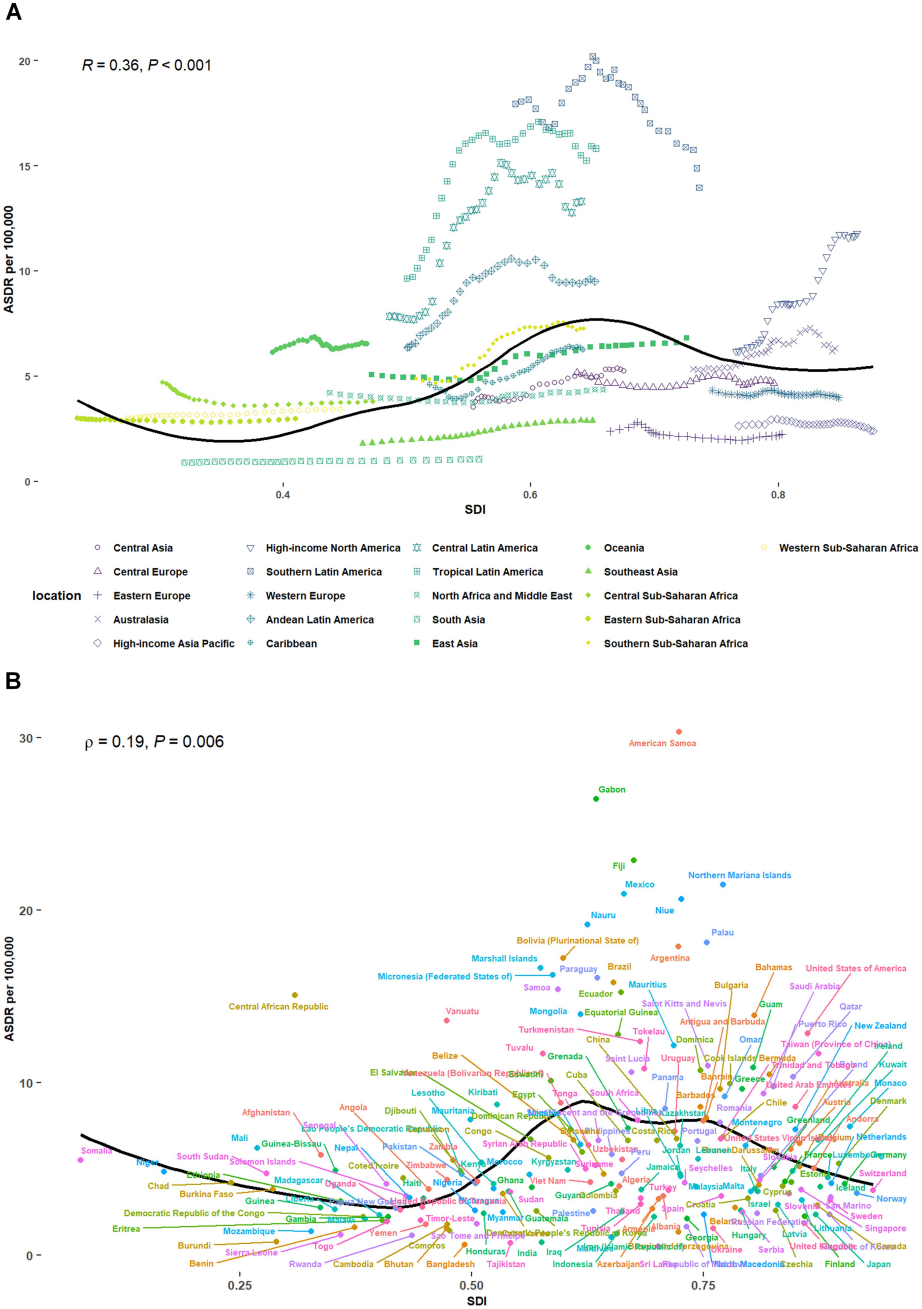
CKD-related ASDR attributable to high red meat intake across 21 GBD regions and 204 countries, by SDI for both sexes combined, 1990–2021. **(A)** The correlation between CKD-related ASDR attributable to high red meat intake and SDI across 21 GBD regions. **(B)** The correlation between CKD-related ASDR attributable to high red meat intake and SDI across 204 countries. CKD, chronic kidney disease; DALYs, disability-adjusted life years; ASDR, age-standardized DALYs rate; GBD, Global Burden of Disease; SDI, socio-demographic index.

Across 21 GBD regions, we investigated the association between SDI and the disease burden of CKD from 1990 to 2021. ASDR showed a slightly positive correlation with SDI (*ρ* = 0.20, *p* = 0.004) and ASMR presented a slightly positive correlation with SDI (*ρ* = 0.16, *p* = 0.019) (Figure 2A; Supplementary Figure 2A). Briefly, as SDI increases, ASDR first raised and then felled, showing a trend similar to an inverted U-shape.

Across 204 countries, our results demonstrated that there was also a slightly positive association between ASDR and SDI (*ρ* = 0.19, *p* = 0.006), with some exceptions. Similar patterns were observed for ASMR in relation to SDI (*ρ* = 0.18, *p* = 0.01) (Figure 2B; Supplementary Figure 2B).

### 3.3 Decomposition analysis

**Figure 3:**
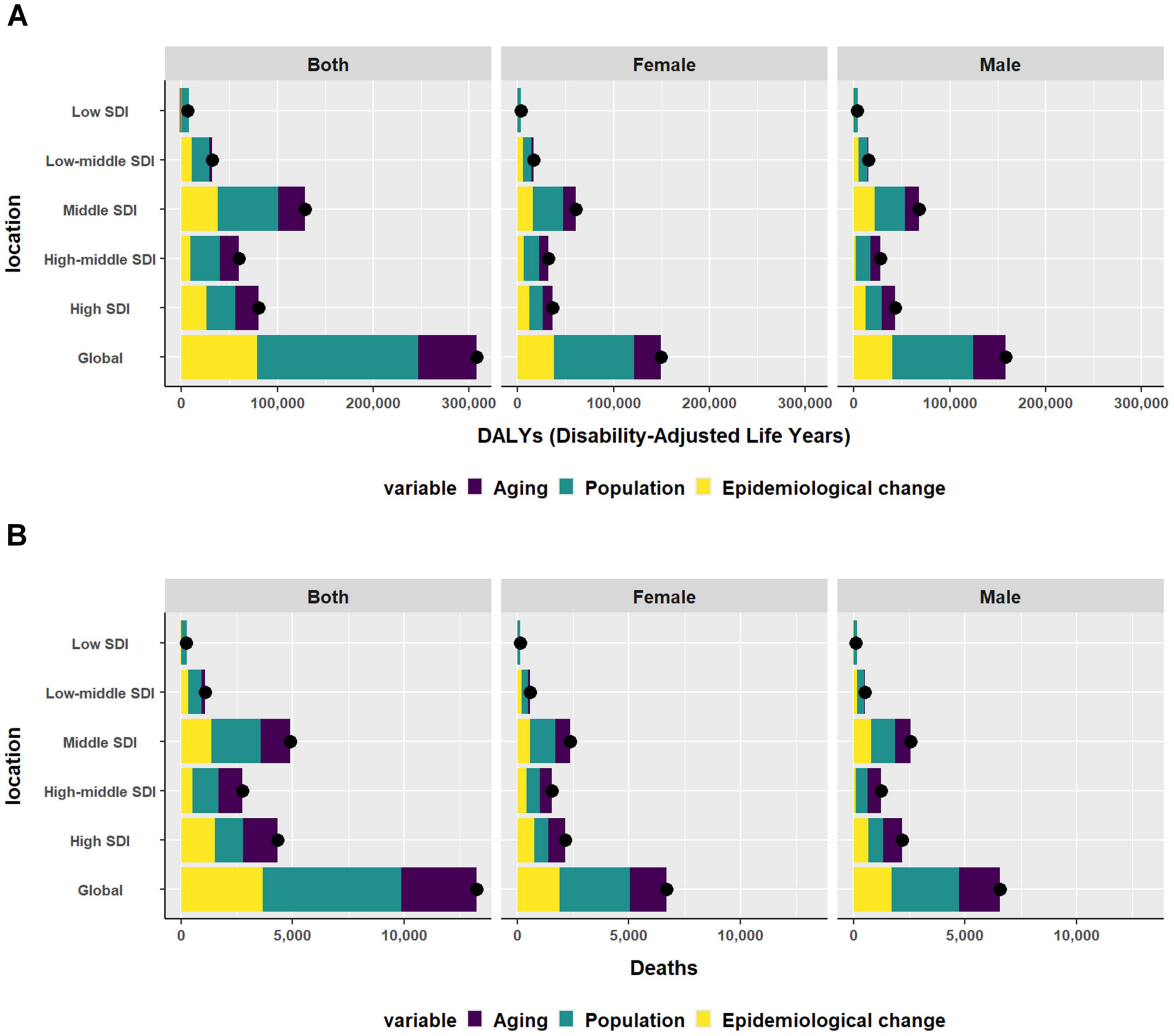
Changes in **(A)** DALYs and **(B)** Deaths of CKD according to aging, and epidemiological change and population from 1990 to 2021 at global level by SDI quintile and by sex. The black dot represents the overall value of the change resulting from all three factors. DALYs, disability-adjusted life-years; CKD, Chronic Kidney Disease; SDI, sociodemographic index.

At the global level and across five SDI quintiles, population was the largest contributor to the DALYs. From 1990 to 2021, the contribution of population was highest in the low SDI region. Aging had the most significant influence in the high-middle SDI region. The contribution of aging was negative in the low SDI region. Epidemiological change had the most significant influence in the low-middle SDI region. Conversely, the contribution of epidemiological change was negative in the low SDI region (Figure 3A). Globally, population drove the death burden, except in the high SDI quintile, where 35.66% of changes were due to aging. This trend held for males, but in females, epidemiological changes were the main cause in the high SDI quintile, followed by aging (Figure 3B).

### 3.4 Global trends by age and sex

**Figure 4:**
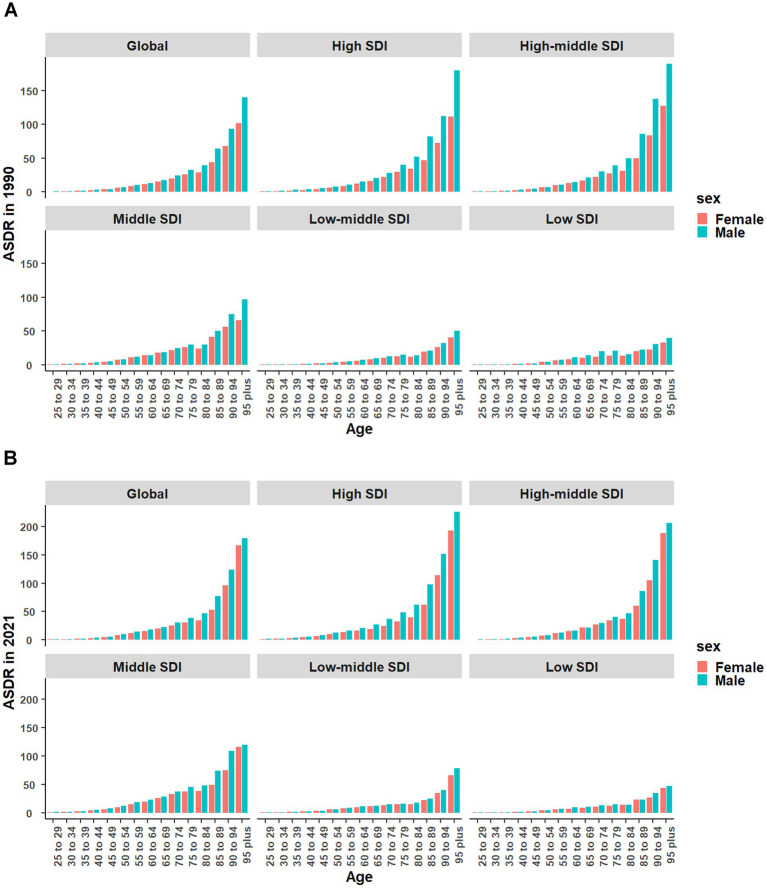
Changes in ASDR of CKD due to high red meat intake from 1990 to 2021 by sex in different SDI quintiles. **(A)** Changes in ASDR of CKD due to high red meat intake in 1990 by sex in different SDI quintiles. **(B)** Changes in ASDR of CKD due to high red meat intake in 2021 by sex in different SDI quintiles. ASDR, age-standardized DALYs rate; DALYs, disability-adjusted life-years; CKD, Chronic Kidney Disease; SDI, sociodemographic index.

Globally, the ASMR and ASDR increased with age, peaking in those 95 plus. Both ASMR and ASDR rose in males and females from 1990 to 2021. Across most age groups, ASDR and ASMR were higher for males, but the gender gap narrowed from 1990 to 2021 (Figure 4; Supplementary Figure 3).

### 3.5 Causal attribution

**Figure 5:**
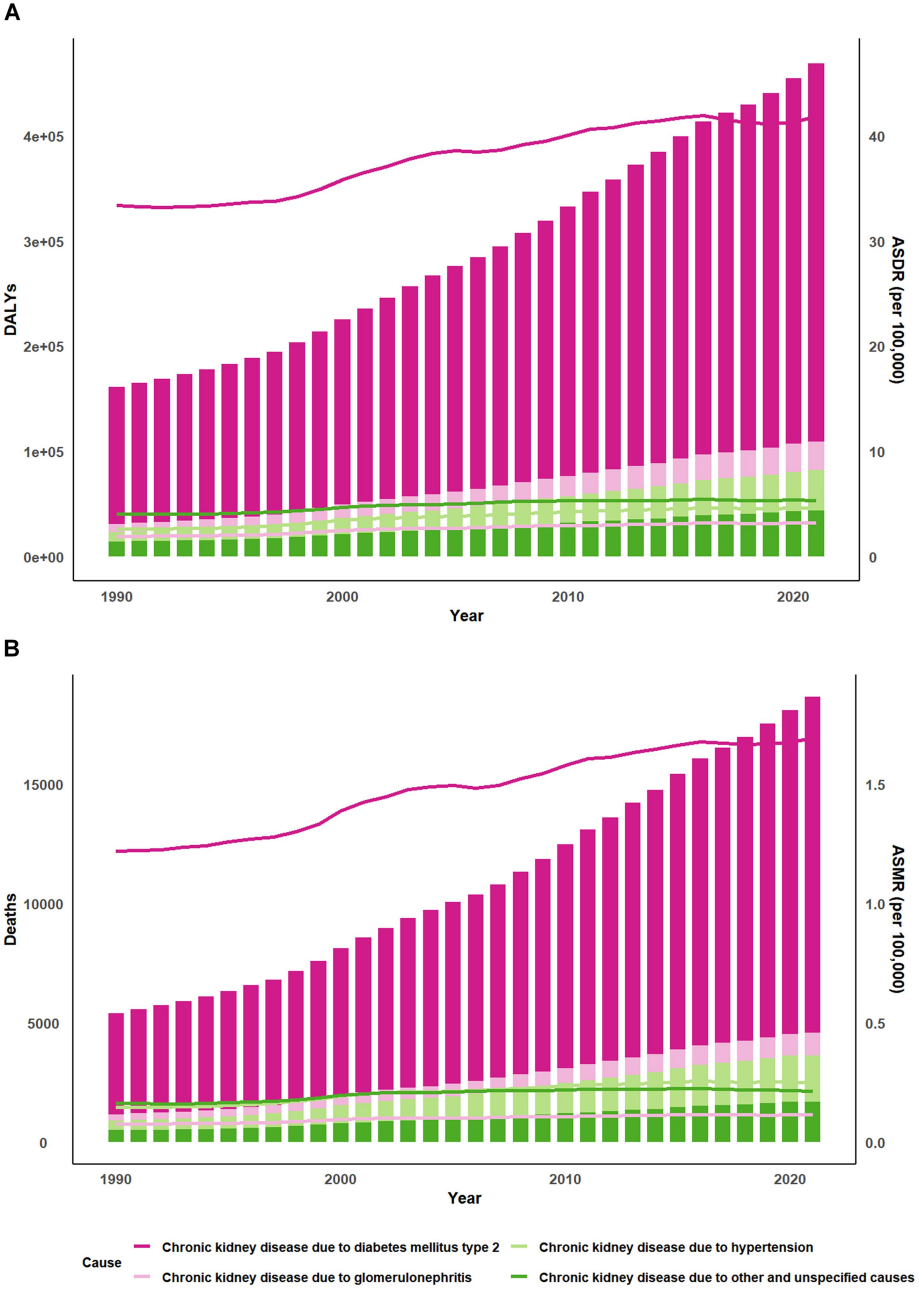
Trends of global burden of CKD due to high red meat intake from 1990 to 2021. Bar charts display the **(A)** DALYs and **(B)** Deaths while line graphs illustrate the age-standardized rates of these measures. CKD, chronic kidney disease; DALYs, disability-adjusted life-years; ASDR, age-standardized DALYs rate; ASMR, age-standardized mortality rate.

Apart from type 1 diabetes mellitus, CKD caused by glomerulonephritis, hypertension, T2DM, and other unspecified causes has all been associated with high red meat. High red meat can lead to an increase in global CKD levels, primarily through T2DM (Figure 5). Further, the decomposition analyses of the DALYs burden due to T2DM indicated that 57.21% of the changes were attributable to population, followed by epidemiological changes and aging globally. Aging had the most significant influence in high-middle SDI region. Epidemiological change had the most significant influence in the high SDI region (Supplementary Table 4). The decomposition analyses of the CKD deaths burden due to T2DM indicated that 48.34% of the changes were attributable to population, followed by epidemiological changes (26.88%) and aging globally. In contrast, for the high SDI quintile, 34.86% of the changes were due to epidemiologie, 34.57% to aging, and 30.57% to population (Supplementary Table 5).

### 3.6 CKD burden in China and the USA

In China, between 1990 and 2021, both ASDR and ASMR rose. Males consistently had higher rates than females, and this pattern was mirrored in the EAPCs. The most notable increases in ASDR and ASMR were linked to glomerulonephritis (Supplementary Tables 6, 7). In the USA, ASDR also increased, with males having higher rates than females. However, ASMR showed a different trend, increasing overall but with females surpassing males by 2021. The EAPCs for males remained higher than that for females. Glomerulonephritis caused the most significant changes in ASMR, while hypertension drove ASDR increases (Supplementary Tables 8, 9). In these two countries, the ASMR and ASDR both increased with age, peaking in those 95+.

## 4 Discussion

Our study comprehensively analyzed the global burden of CKD attributable to increased red meat consumption from 1990 to 2021, stratified by year, age, sex, region, and SDI. Our findings indicated that, globally, the DALYs and deaths of CKD attributed to high red meat consumption remained substantial and significantly increased from 1990 to 2021. This suggests that the burden of CKD due to increased red meat consumption continues to be a major global public health issue.

According to the GBD 2019, CKD ranked 12th among the 133 leading causes of death in the GBD Study (19), with nearly 700 million CKD patients globally, surpassing the numbers for chronic diseases like diabetes and chronic obstructive pulmonary disease (2, 20). This study focused on the burden of CKD attributable to increased red meat consumption, suggesting an underestimation of the potential burden of CKD caused by excessive red meat consumption.

The burden of disease is closely linked to socioeconomic development. In our study, we found that at both regional and national levels, as SDI increased, both ASMR and ASDR also rose, aligning with the levels of red meat consumption across different regions and countries. High-income countries consumed about five times more red meat than low-income countries (21). Although red meat consumption has decreased in high-income countries over the past decade, the overall consumption remains higher than in low-income countries. Previous study showed that high SDI region and countries exhibited a lower CKD burden caused by high blood sugar (22). However, our findings indicated the opposite, with SDI positively correlated with ASMR and ASDR, and high SDI region experiencing the most significant growth in mortality burden. This suggests that the CKD burden due to excessive red meat consumption has not been fully recognized by the public, warranting further development of public health measures to mitigate this trend. Additionally, a potential explanation for this trend is the difficulty in conducting comprehensive CKD screening in areas with limited medical facilities and laboratory diagnostic services, leading to underreporting of CKD data in low SDI region. Therefore, we should interpret these results cautiously and avoid viewing the association between red meat-attributed CKD burden and SDI as simple or linear.

At the regional level, SLA and TLA had the highest ASMR and ASDR, consistent with previous research findings (22, 23). In contrast, South Asian had the lowest ASMR and ASDR, which may be influenced by cultural or religious dietary habits in these countries. For instance, in predominantly Buddhist South Asian countries like India, Sri Lanka, and Bangladesh, diets typically prioritize vegetables, legumes, grains, and dairy products over red meat. In India, Hindu reverence for cows prohibits their slaughter, resulting in minimal beef consumption (24).

Recent investigations have revealed that, in addition to a high red meat diet, high body mass index (BMI) and high fasting plasma glucose are also significant contributors to the increasing burden of CKD. However, there are notable differences in the relative contributions of these risk factors across different regions and diseases. Tan et al. examined the global burden of CKD attributable to high BMI using GBD 2021 data and confirmed that the global burden of CKD attributable to high BMI has risen markedly over the past three decades, particularly in high-SDI regions (25). Besides, recent studies have shown that while the proportion of deaths and DALYs attributed to high BMI has increased globally, the contributions of high fasting plasma glucose have shown a smaller upward trend (25–28). In all, our study demonstrates a significant global burden of CKD attributable to high red meat consumption, with distinct geographical and demographic patterns. These findings align with and diverge from other GBD-based studies on dietary and metabolic risk factors, such as high BMI and high fasting plasma glucose, highlighting both shared and unique public health challenges.

We further employed decomposition analysis to explicitly disentangle the contributions of demographic drivers from true epidemiological shifts to the changing burden of CKD attributable to red meat consumption. The analysis clearly distinguished that for DALYs, the burden in the high-middle SDI region was primarily driven by demographic aging. In contrast, the low-middle SDI region was most affected by an epidemiological shift (reflected in changing ASMR), while the low SDI region’s burden was predominantly driven by population growth. A similar pattern was observed for mortality, where the high SDI region faced the largest demographic driver of aging, while population growth was the key demographic driver in other regions. These findings logically align with broader population trends: severe aging in high-middle SDI regions and rapid growth in low SDI regions. Therefore, while the risk factor (red meat) is epidemiological, its resulting burden is simultaneously modulated by independent demographic forces. This underscores that public health measures should be tailored with different focuses for regions with varying SDI levels.

The results of this study showed that for individuals aged 25 and above, both the ASMR and ASDR attributable to red meat consumption increased with age in both men and women, reaching their peak at the age of 95. This suggests that older adults are at higher risk for CKD, with a higher prevalence of the disease, which is consistent with previous research findings (23). From 1990 to 2021, men have consistently experienced a higher burden of CKD attributable to red meat consumption. However, the gender gap has gradually narrowed over time, particularly in the USA. This phenomenon may be attributed to higher red meat consumption among men, but with socio-economic progress, the economic and social status gap between men and women has gradually narrowed (29), leading to an increase in women’s red meat consumption, thus reducing the gender difference in CKD burden.

We further explored the cause of CKD due to increased red meat consumption and found that T2DM was the main cause. Consuming large amounts of red meat leads to increased intake of saturated fats, cholesterol, iron, and salt, as well as excessive acid load. It may also result in the gut microbiota producing higher levels of uremic toxins, such as trimethylamine N-oxide, indole sulfate, and p-cresyl sulfate. These metabolic disturbances and uremic toxins are closely associated with an increased risk of T2DM (5). Previous studies have confirmed that red meat consumption is associated with an increased risk of T2DM, with a stronger correlation in Western environments and no gender differences (30–32). Moreover, even without considering the quantity, cooking red meat with direct flame or at high temperatures was also associated with an increased risk of T2DM (33, 34).

The highest number of CKD deaths due to high red meat intake occurred in the USA, followed by China, a result that caught our attention. Decomposition analysis indicated that the main effect on both ASDR and ASMR in both SDI regions was aging. China and the USA rank first and third in global population size, respectively, which suggests that both a large population base and aging could contribute to the two countries having the highest red meat intake associated CKD death tolls worldwide.

This study has certain limitations. We used the GBD 2021 database, which primarily consists of national and regional data without individual-level data, making it impossible to validate our conclusions on a personal level. Besides, GBD 2021 does not include dietary survey results, thus it does not establish a causal relationship between red meat consumption and CKD. Lastly, individuals who consume high amounts of red meat may also have other unhealthy lifestyle habits, such as low vegetable and fruit consumption, lack of exercise, smoking, and other confounding factors such as overweight status and lower education levels. These confounders may influence the relationship between red meat consumption and CKD.

## 5 Conclusion

Between 1990 and 2021, CKD caused by high red meat consumption led to a continuous rise in global ASDR and ASMR, with high and middle SDI regions bearing the brunt of the burden. Older adults and men exhibited a higher disease burden, however, the gender gap has gradually narrowed over time. In light of these findings, governments worldwide should strengthen education on diet, particularly in high and middle SDI regions to reduce the CKD burden caused by increased red meat consumption.

## Data Availability

The original contributions presented in the study are included in the article/Supplementary material, further inquiries can be directed to the corresponding author.

## Footnotes

^1^ http://ghdx.healthdata.org/gbd-results-tool
