# Supplementary material for: Global, regional, and national burden of chronic kidney disease attributable to red meat consumption from 1990 to 2021
Source: Front Nutr. 2025 Sep 19;12:1666684. doi: 10.3389/fnut.2025.1666684 (PMC12491058; doi:10.3389/fnut.2025.1666684)
Supplement: Supplementary file 1 [file Table_1.DOCX]

Supplementary Material

# Supplementary Figures and Tables

**Supplementary Table 1:** The cases, ASMR and EAPC of CKD Deaths from 1990 to 2021 globally.

| **Deaths** | **1990** | | **2021** | | **EAPC in ASMR** **(95% CI)** |
| --- | --- | --- | --- | --- | --- |
|  | **Cases (95% UI)** | **ASMR/100000 (95% UI)** | **Cases (95% UI)** | **ASMR/100000 (95% UI)** |  |
| Global | 5397.37 (0.28 to 12155.36) | 0.16 (0 to 0.36) | 18652.37 (0.45 to 40456.22) | 0.23 (0 to 0.5) | 1.33 (1.21 to 1.45) |
| **Sex** |  |  |  |  |  |
| Male | 2645.97 (0.15 to 6073.28) | 0.19 (0 to 0.43) | 9213 (0.24 to 20205.81) | 0.26 (0 to 0.58) | 1.25 (1.16 to 1.35) |
| Female | 2751.4 (0.14 to 6409.39) | 0.14 (0 to 0.33) | 9439.37 (0.19 to 20962.5) | 0.2 (0 to 0.45) | 1.3 (1.16 to 1.44) |
| **SDI** |  |  |  |  |  |
| High SDI | 1811.15 (0.15 to 4128.4) | 0.17 (0 to 0.38) | 6118.18 (0.19 to 13658.2) | 0.26 (0 to 0.57) | 1.72 (1.59 to 1.85) |
| High-middle SDI | 1505.28 (0.11 to 3402.6) | 0.18 (0 to 0.41) | 4263.46 (0.11 to 9367.21) | 0.22 (0 to 0.49) | 0.74 (0.61 to 0.87) |
| Middle SDI | 1476.19 (0.02 to 3325.94) | 0.17 (0 to 0.39) | 6381.56 (0.08 to 13943.66) | 0.26 (0 to 0.57) | 1.54 (1.38 to 1.71) |
| Low-middle SDI | 397.37 (0.01 to 926) | 0.08 (0 to 0.18) | 1465.8 (0.02 to 3204.73) | 0.11 (0 to 0.25) | 1.28 (1.13 to 1.42) |
| Low-SDI | 200.78 (0 to 480.13) | 0.11 (0 to 0.25) | 409.35 (0.01 to 979.21) | 0.1 (0 to 0.23) | -0.32 (-0.45 to -0.2) |
| **GBD region** |  |  |  |  |  |
| Andean Latin America | 52.47 (0 to 118.54) | 0.28 (0 to 0.63) | 231.02 (0 to 517.26) | 0.4 (0 to 0.9) | 1.27 (0.86 to 1.67) |
| Australasia | 47.95 (0 to 110.16) | 0.22 (0 to 0.51) | 176.87 (0 to 414.12) | 0.28 (0 to 0.66) | 1.5 (1.22 to 1.78) |
| Caribbean | 42.1 (0 to 94.07) | 0.17 (0 to 0.39) | 134.62 (0 to 299.07) | 0.25 (0 to 0.55) | 1.93 (1.67 to 2.2) |
| Central Asia | 24.1 (0 to 59.23) | 0.05 (0 to 0.13) | 96.82 (0.02 to 227.7) | 0.12 (0 to 0.29) | 2.61 (2.31 to 2.9) |
| Central Europe | 224.36 (0.01 to 507.85) | 0.16 (0 to 0.36) | 329.35 (0 to 755.57) | 0.14 (0 to 0.33) | -0.2 (-0.42 to 0.02) |
| Central Latin America | 217.71 (0 to 503.35) | 0.29 (0 to 0.66) | 1145.29 (0 to 2663.98) | 0.46 (0 to 1.06) | 2.04 (1.47 to 2.62) |
| **Central Sub-Saharan Africa** | **33.78 (0 to 77.36)** | **0.19 (0 to 0.44)** | **69.28 (0 to 158.33)** | **0.15 (0 to 0.35)** | **-0.81 (-1 to -0.61)** |
| East Asia | 1403.1 (0.01 to 3180.8) | 0.21 (0 to 0.5) | 5621.39 (0.08 to 12420.61) | 0.28 (0 to 0.62) | 1.04 (0.89 to 1.19) |
| Eastern Europe | 97.81 (0.03 to 234.75) | 0.04 (0 to 0.09) | 213.43 (0.01 to 509.12) | 0.06 (0 to 0.14) | 1.03 (0.53 to 1.54) |
| Eastern Sub-Saharan Africa | 83.52 (0 to 200.87) | 0.14 (0 to 0.33) | 188.24 (0 to 450.77) | 0.14 (0 to 0.33) | 0.07 (0.01 to 0.12) |
| High-income Asia Pacific | 213.3 (0 to 505.86) | 0.12 (0 to 0.3) | 711.88 (0 to 1684.05) | 0.11 (0 to 0.26) | -0.52 (-0.65 to -0.39) |
| High-income North America | 694.43 (0.07 to 1572.81) | 0.19 (0 to 0.44) | 3073.36 (0.11 to 6830.45) | 0.45 (0 to 0.99) | 2.96 (2.75 to 3.18) |
| North Africa and Middle East | 251.27 (0 to 586.21) | 0.18 (0 to 0.44) | 741.38 (0 to 1722.63) | 0.19 (0 to 0.44) | 0.22 (0.01 to 0.42) |
| Oceania | 5.67 (0 to 13.61) | 0.23 (0 to 0.54) | 16.62 (0 to 38.15) | 0.27 (0 to 0.61) | 0.26 (0.14 to 0.38) |
| South Asia | 156.56 (0 to 368) | 0.03 (0 to 0.08) | 492.1 (0.01 to 1181.92) | 0.04 (0 to 0.09) | 0.21 (0.11 to 0.32) |
| Southeast Asia | 154.99 (0 to 366.34) | 0.07 (0 to 0.17) | 688.78 (0 to 1546.5) | 0.12 (0 to 0.27) | 1.71 (1.64 to 1.78) |
| Southern Latin America | 348.54 (0.01 to 757.44) | 0.81 (0 to 1.74) | 580.09 (0 to 1268.41) | 0.64 (0 to 1.39) | -0.36 (-0.72 to 0) |
| Southern Sub-Saharan Africa | 38.26 (0 to 92.12) | 0.16 (0 to 0.37) | 131.1 (0 to 294.68) | 0.26 (0 to 0.58) | 2.09 (1.79 to 2.4) |
| Tropical Latin America | 282.27 (0.01 to 607.3) | 0.35 (0 to 0.78) | 1485.2 (0.03 to 3215.88) | 0.59 (0 to 1.27) | 1.65 (1.23 to 2.08) |
| Western Europe | 938.41 (0.08 to 2163.76) | 0.16 (0 to 0.37) | 2305.3 (0.06 to 5308.36) | 0.18 (0 to 0.42) | 0.83 (0.66 to 0.99) |
| Western Sub-Saharan Africa | 86.78 (0 to 211.48) | 0.12 (0 to 0.28) | 220.25 (0 to 531.16) | 0.14 (0 to 0.33) | 0.4 (0.35 to 0.45) |

ASMR=age standardised mortality rate; EAPC=estimated annual percentage change; CKD=chronic kidney disease; UI=uncertainty interval; CI=confidence interval; SDI=sociodemographic index.

**Supplementary Table 2:** The cases, ASDR and EAPC of CKD DALYs from 1990 to 2021 globally.

| **DALYs** | **1990** | | **2021** | | | **EAPC in ASDR** **(95% CI)** |
| --- | --- | --- | --- | --- | --- | --- |
|  | **Cases (95% UI)** | **ASDR/100000(95% UI)** | **Cases (95% UI)** | **ASDR/100000(95% UI)** | |  |
| Global | 161297.04 (12.65 to 360914.18) | **4.2 (0 to 9.28)** | 468895.2 (17.42 to 1027391.03) | **5.5 (0 to 12.01)** | 1.07 (0.97 to 1.17) | |
| **Sex** |  |  |  |  | |  |
| Male | 81663.65 (6.78 to 185577.78) | 4.72 (0 to 10.68) | 239854.77 (8.8 to 520422.82) | 6.12 (0 to 13.38) | | 1.08 (0.99 to 1.17) |
| Female | 79633.39 (5.32 to 178517.12) | 3.82 (0 to 8.55) | 229040.43 (9.78 to 510972.41) | 4.99 (0 to 11.18) | | 1.01 (0.9 to 1.12) |
| **SDI** |  |  |  |  | |  |
| High SDI | 51688.34 (6.81 to 114676) | 4.77 (0 to 10.61) | 131885.57 (8.09 to 282414.51) | 6.59 (0 to 14.33) | | 1.3 (1.19 to 1.41) |
| High-middle SDI | 45069.13 (5.04 to 102887.91) | 4.77 (0 to 10.75) | 105037.14 (5.55 to 230075.5) | 5.43 (0 to 11.92) | | 0.54 (0.44 to 0.65) |
| Middle SDI | 46113.28 (0.96 to 103189.71) | 4.42 (0 to 9.85) | 174669.95 (3.44 to 384704.2) | 6.5 (0 to 14.21) | | **1.52 (1.33 to 1.7)** |
| Low-middle SDI | 12205.72 (0.4 to 28831.95) | 2.02 (0 to 4.74) | 44232.74 (1.24 to 98203.67) | 2.99 (0 to 6.62) | | 1.36 (1.22 to 1.51) |
| Low-SDI | 6014.64 (0.14 to 14491.56) | 2.63 (0 to 6.3) | 12705.53 (0.23 to 30950.87) | 2.38 (0 to 5.66) | | -0.37 (-0.47 to -0.27) |
| **GBD region** |  |  |  |  | |  |
| Andean Latin America | 1306.2 (0.01 to 2907.33) | 6.37 (0 to 14.36) | 5703.08 (0.07 to 12531.94) | 9.51 (0 to 21) | | 1.44 (1.05 to 1.82) |
| Australasia | 1216.99 (0.1 to 2776.71) | 5.35 (0 to 12.19) | 3508.72 (0.06 to 8015.09) | 6.34 (0 to 14.45) | | 0.95 (0.75 to 1.15) |
| Caribbean | 1207.73 (0 to 2659.22) | 4.61 (0 to 10.08) | 3371.8 (0 to 7500.47) | 6.27 (0 to 13.92) | | 1.75 (1.51 to 1.98) |
| Central Asia | 1724.1 (0.17 to 4233.46) | 3.53 (0 to 8.57) | 4645.05 (0.8 to 11088.83) | 5.24 (0 to 12.39) | | 1.37 (1.2 to 1.54) |
| Central Europe | 7477.87 (0.36 to 16866.71) | 5.17 (0 to 11.71) | 9592 (0.08 to 21637.12) | 4.67 (0 to 10.56) | | 0.02 (-0.15 to 0.18) |
| Central Latin America | 7016.62 (0.03 to 16084.25) | 7.84 (0 to 17.95) | 34490.44 (0.08 to 78310.5) | 13.31 (0 to 30.28) | | 2.2 (1.66 to 2.74) |
| Central Sub-Saharan Africa | 1108.79 (0.03 to 2519.02) | 4.71 (0 to 10.68) | 2374.25 (0.02 to 5453.95) | 3.82 (0 to 8.68) | | -0.74 (-0.91 to -0.56) |
| East Asia | 42256.87 (0.19 to 94189.46) | 5.07 (0 to 11.52) | 147084.23 (4.21 to 313616.5) | 6.81 (0 to 14.53) | | 1.26 (1.09 to 1.44) |
| **Eastern Europe** | **6423.45 (1.64 to 15493.32)** | **2.39 (0 to 5.76)** | **7679.22 (0.46 to 18860.28)** | **2.25 (0 to 5.61)** | | **-0.79 (-1.04 to -0.53)** |
| Eastern Sub-Saharan Africa | 2176.68 (0.02 to 5232.16) | 2.99 (0 to 7.18) | 4860.36 (0.07 to 11468) | 2.96 (0 to 7.01) | | -0.11 (-0.16 to -0.05) |
| High-income Asia Pacific | 5036.71 (0.13 to 11609.11) | 2.63 (0 to 6.04) | 12164.77 (0.1 to 28589.57) | 2.39 (0 to 5.59) | | -0.26 (-0.42 to -0.11) |
| High-income North America | 21066.39 (3.49 to 46434.87) | 6.18 (0 to 13.68) | 70922.24 (4.73 to 151440.38) | 11.78 (0 to 25) | | 2.37 (2.18 to 2.55) |
| North Africa and Middle East | 7114.88 (0.15 to 17252.68) | 4.21 (0 to 9.96) | 20873.61 (0.03 to 48070.22) | 4.38 (0 to 9.99) | | 0.25 (0.09 to 0.41) |
| Oceania | 191.25 (0 to 456.21) | 6.13 (0 to 14.3) | 498.25 (0.01 to 1155.94) | 6.53 (0 to 14.99) | | -0.02 (-0.12 to 0.08) |
| South Asia | 5228.41 (0.16 to 12875.3) | 0.9 (0 to 2.17) | 16422.52 (0.55 to 39407.71) | 1.07 (0 to 2.55) | | 0.46 (0.4 to 0.52) |
| Southeast Asia | 4554.43 (0.01 to 10800.97) | 1.8 (0 to 4.26) | 19288.62 (0.05 to 44519.75) | 2.88 (0 to 6.62) | | 1.72 (1.63 to 1.8) |
| Southern Latin America | 8183.41 (0.49 to 17638.47) | 17.94 (0 to 38.61) | 12082.39 (0.07 to 26447.19) | 13.96 (0 to 30.62) | | -0.48 (-0.76 to -0.19) |
| Southern Sub-Saharan Africa | 1436.68 (0.04 to 3333.47) | 4.9 (0 to 11.63) | 4529.7 (0.05 to 10177) | 7.28 (0 to 15.94) | | 1.73 (1.46 to 2) |
| Tropical Latin America | 9274.19 (0.27 to 20478.08) | 9.64 (0 to 20.94) | 41022.58 (1.45 to 86880.67) | 15.82 (0 to 33.53) | | 1.48 (1.03 to 1.93) |
| Western Europe | 24655.35 (3.8 to 55243.03) | 4.32 (0 to 9.79) | 40558.59 (2.76 to 92898.08) | 3.97 (0 to 9.16) | | -0.11 (-0.18 to -0.04) |
| Western Sub-Saharan Africa | 2640.03 (0.03 to 6482.6) | 3 (0 to 7.23) | 7222.81 (0.08 to 17311.08) | 3.42 (0 to 8.21) | | 0.41 (0.37 to 0.44) |

ASDR=age-standardised DALYs rate; EAPC=estimated annual percentage change; CKD=chronic kidney disease; UI=uncertainty interval; CI=confidence interval; SDI=sociodemographic index.

**Supplementary Table 3:** The case number and ASR of DALYs and Deaths due to CKD caused by red meat consumption in 2021, by countries/territories.

| **Countries** | **DALYs cases** **(95% UI)** | **ASDR/100000** **(95% UI)** | **Deaths cases** **(95% UI)** | **ASMR/100000** **(95% UI)** |  |
| --- | --- | --- | --- | --- | --- |
|  |  |  |  |  |  |
| Afghanistan | 664.02 (0 to 1819.99) | 5.8 (0 to 15.57) | 20.37 (0 to 57.05) | 0.07 (0 to 0.18) |  |
| Albania | 143.22 (0 to 349.31) | 3.42 (0 to 8.41) | 5.03 (0 to 12.56) | 0.19 (0 to 0.47) |  |
| Algeria | 1409.89 (0 to 3298.08) | 4.01 (0 to 9.29) | 53.32 (0 to 129.65) | 0.12 (0 to 0.29) |  |
| American Samoa | 14.44 (0 to 33.31) | 30.36 (0 to 69.89) | 0.58 (0 to 1.35) | 1.16 (0 to 2.71) |  |
| Andorra | 8.09 (0 to 17.92) | 5.03 (0 to 11.1) | 0.37 (0 to 0.85) | 0.43 (0 to 0.99) |  |
| Angola | 482.89 (0 to 1201.27) | 3.83 (0 to 9.12) | 14.37 (0 to 34.41) | 0.04 (0 to 0.11) |  |
| Antigua and Barbuda | 8.39 (0 to 18.14) | 7.8 (0 to 16.93) | 0.33 (0 to 0.73) | 0.37 (0 to 0.81) |  |
| Argentina | 9935.15 (0 to 21720) | 17.89 (0 to 39.14) | 474.26 (0 to 1055.56) | 1.04 (0 to 2.32) |  |
| Armenia | 135.04 (0 to 320.17) | 3.26 (0 to 7.78) | 3.34 (0 to 8.04) | 0.11 (0 to 0.27) |  |
| Australia | 2909.46 (0 to 6656.11) | 6.14 (0 to 14.13) | 150.36 (0 to 354.06) | 0.58 (0 to 1.37) |  |
| Austria | 1350.34 (0.01 to 3023.94) | 6.49 (0 to 14.51) | 85.64 (0 to 199.13) | 0.95 (0 to 2.22) |  |
| Azerbaijan | 298.48 (0 to 739.05) | 2.68 (0 to 6.51) | 5.73 (0 to 14.2) | 0.05 (0 to 0.14) |  |
| Bahamas | 60 (0 to 135.15) | 13.89 (0 to 31.41) | 1.96 (0 to 4.41) | 0.51 (0 to 1.14) |  |
| Bahrain | 67.19 (0 to 157.04) | 7.95 (0 to 18.16) | 2.24 (0 to 5.12) | 0.15 (0 to 0.33) |  |
| Bangladesh | 865.48 (0 to 1973.62) | 0.62 (0 to 1.41) | 26.09 (0 to 59.42) | 0.02 (0 to 0.04) |  |
| Barbados | 41.37 (0 to 97.25) | 8.61 (0 to 20.25) | 1.8 (0 to 4.17) | 0.6 (0 to 1.4) |  |
| Belarus | 426.9 (0.03 to 1023.14) | 2.75 (0 to 6.72) | 5.15 (0 to 11.95) | 0.06 (0 to 0.13) |  |
| Belgium | 1255.8 (0.1 to 2842.61) | 5.13 (0 to 11.82) | 60.21 (0 to 144.56) | 0.52 (0 to 1.26) |  |
| Belize | 22.13 (0 to 48.84) | 6.67 (0 to 14.95) | 0.75 (0 to 1.7) | 0.18 (0 to 0.4) |  |
| Benin | 82.03 (0 to 197.48) | 1.6 (0 to 3.81) | 2.96 (0 to 7.05) | 0.02 (0 to 0.05) |  |
| Bermuda | 13.15 (0 to 29.21) | 10.48 (0 to 23.02) | 0.6 (0 to 1.35) | 0.94 (0 to 2.12) |  |
| Bhutan | 9.87 (0 to 23.01) | 1.54 (0 to 3.61) | 0.32 (0 to 0.8) | 0.04 (0 to 0.11) |  |
| Bolivia (Plurinational State of) | 1590.34 (0 to 3724.16) | 17.22 (0 to 39.69) | 62.55 (0 to 142.7) | 0.53 (0 to 1.21) |  |
| Bosnia and Herzegovina | 79.61 (0 to 187.83) | 1.35 (0 to 3.17) | 2.95 (0 to 7.14) | 0.09 (0 to 0.22) |  |
| Botswana | 72.87 (0 to 169.7) | 4.68 (0 to 10.91) | 2.08 (0 to 4.86) | 0.09 (0 to 0.2) |  |
| Brazil | 40043.43 (1.43 to 84285.15) | 15.81 (0 to 33.31) | 1450.33 (0.03 to 3140.15) | 0.66 (0 to 1.43) |  |
| Brunei Darussalam | 12.98 (0 to 31.09) | 4.06 (0 to 9.61) | 0.48 (0 to 1.15) | 0.11 (0 to 0.25) |  |
| Bulgaria | 1205.6 (0 to 2883.21) | 9.64 (0 to 23.5) | 43.99 (0 to 104.3) | 0.65 (0 to 1.54) |  |
| Burkina Faso | 352.8 (0 to 847.23) | 3.81 (0 to 8.98) | 12.37 (0 to 29.19) | 0.05 (0 to 0.13) |  |
| Burundi | 34.85 (0 to 86.22) | 0.78 (0 to 1.91) | 1.39 (0 to 3.39) | 0.01 (0 to 0.03) |  |
| Cabo Verde | 17.49 (0 to 43.27) | 3.55 (0 to 8.88) | 0.58 (0 to 1.45) | 0.1 (0 to 0.26) |  |
| Cambodia | 219.73 (0 to 532.18) | 1.78 (0 to 4.33) | 7.48 (0 to 18.89) | 0.04 (0 to 0.11) |  |
| Cameroon | 733.98 (0 to 1884.77) | 5.5 (0 to 13.73) | 23.31 (0 to 57.93) | 0.07 (0 to 0.18) |  |
| Canada | 2057.71 (0 to 4722.44) | 2.89 (0 to 6.51) | 106.06 (0 to 243.12) | 0.28 (0 to 0.65) |  |
| Central African Republic | 416.09 (0 to 1024.04) | 15.07 (0 to 36.56) | 10.86 (0 to 26.4) | 0.2 (0 to 0.48) |  |
| Chad | 258.35 (0 to 660.21) | 4.19 (0 to 10.37) | 8.02 (0 to 20.76) | 0.05 (0 to 0.12) |  |
| Chile | 1735.36 (0 to 3936.7) | 6.83 (0 to 15.56) | 82.55 (0 to 191.69) | 0.44 (0 to 1.02) |  |
| **China** | **141230.18 (4.21 to 302598.07)** | **6.76 (0 to 14.42)** | **5365.8 (0.07 to 11824.07)** | **0.38 (0 to 0.83)** |  |
| Colombia | 2084.8 (0 to 4916.58) | 3.73 (0 to 8.85) | 72.96 (0 to 178.35) | 0.15 (0 to 0.36) |  |
| Comoros | 6.61 (0 to 16.55) | 1.43 (0 to 3.53) | 0.28 (0 to 0.71) | 0.04 (0 to 0.09) |  |
| Congo | 170.63 (0 to 423.63) | 5.64 (0 to 13.6) | 5.15 (0 to 12.67) | 0.1 (0 to 0.23) |  |
| Cook Islands | 2.53 (0 to 5.78) | 9.94 (0 to 23.07) | 0.11 (0 to 0.25) | 0.61 (0 to 1.44) |  |
| Costa Rica | 368.57 (0 to 851.95) | 6.64 (0 to 15.32) | 12.95 (0 to 29.84) | 0.27 (0 to 0.63) |  |
| Coted'Ivoire | 570.97 (0 to 1404.42) | 4.47 (0 to 10.62) | 16.59 (0 to 40.09) | 0.06 (0 to 0.14) |  |
| Croatia | 282.23 (0 to 661.65) | 3.28 (0 to 7.82) | 12.97 (0 to 30.6) | 0.31 (0 to 0.73) |  |
| Cuba | 1235.63 (0 to 2811.69) | 6.66 (0 to 15.13) | 49.11 (0 to 109.59) | 0.44 (0 to 0.97) |  |
| Cyprus | 66.58 (0 to 153.88) | 3.98 (0 to 9.3) | 3.26 (0 to 7.56) | 0.24 (0 to 0.56) |  |
| Czechia | 507.6 (0 to 1146.08) | 2.59 (0 to 5.89) | 17.58 (0 to 40.99) | 0.17 (0 to 0.39) |  |
| Democratic People's Republic of Korea | 835.75 (0 to 1978.07) | 2.54 (0 to 5.95) | 28.51 (0 to 65.48) | 0.11 (0 to 0.25) |  |
| Democratic Republic of the Congo | 924.81 (0 to 2226.14) | 2.23 (0 to 5.39) | 26.88 (0 to 65.83) | 0.03 (0 to 0.07) |  |
| Denmark | 667 (0.02 to 1524.96) | 5.29 (0 to 11.99) | 36.4 (0 to 83.32) | 0.62 (0 to 1.42) |  |
| Djibouti | 29.21 (0 to 73.76) | 4.86 (0 to 11.73) | 1.08 (0 to 2.71) | 0.09 (0 to 0.22) |  |
| Dominica | 8.78 (0 to 20.53) | 10.71 (0 to 24.88) | 0.34 (0 to 0.78) | 0.5 (0 to 1.16) |  |
| Dominican Republic | 610.41 (0 to 1409.83) | 5.96 (0 to 13.78) | 22.62 (0 to 53.38) | 0.21 (0 to 0.48) |  |
| Ecuador | 2510.75 (0 to 6116.35) | 15.25 (0 to 36.84) | 97.93 (0 to 231.56) | 0.54 (0 to 1.28) |  |
| Egypt | 4532.62 (0 to 10680) | 7.3 (0 to 16.89) | 165.31 (0 to 387.86) | 0.16 (0 to 0.37) |  |
| El Salvador | 414.66 (0 to 975.7) | 6.71 (0 to 15.75) | 15.51 (0 to 38.13) | 0.24 (0 to 0.59) |  |
| Equatorial Guinea | 83.32 (0 to 208.66) | 12.81 (0 to 31.41) | 2.35 (0 to 5.91) | 0.16 (0 to 0.39) |  |
| Eritrea | 52.58 (0 to 138.42) | 2.02 (0 to 5.26) | 1.91 (0 to 5.04) | 0.03 (0 to 0.08) |  |
| Estonia | 118.67 (0 to 294.24) | 4.23 (0 to 10.59) | 5.56 (0 to 13.5) | 0.42 (0 to 1.03) |  |
| Eswatini | 63.65 (0 to 157.81) | 10.1 (0 to 24.63) | 1.86 (0 to 4.51) | 0.16 (0 to 0.39) |  |
| Ethiopia | 1272.54 (0 to 3049.68) | 3.13 (0 to 7.49) | 55.42 (0 to 134.65) | 0.05 (0 to 0.12) |  |
| Fiji | 169.81 (0 to 401.24) | 22.88 (0 to 53.81) | 6.42 (0 to 14.85) | 0.69 (0 to 1.61) |  |
| Finland | 289.34 (0.02 to 688.17) | 2.25 (0 to 5.33) | 13.3 (0 to 31.11) | 0.24 (0 to 0.56) |  |
| France | 6876.18 (0.31 to 15637.51) | 4.26 (0 to 9.82) | 387.97 (0 to 914) | 0.58 (0 to 1.38) |  |
| Gabon | 296.51 (0 to 721.6) | 26.45 (0 to 63.73) | 9.68 (0 to 23.69) | 0.53 (0 to 1.3) |  |
| Gambia | 23.19 (0 to 55.62) | 2.23 (0 to 5.23) | 0.78 (0 to 1.87) | 0.03 (0 to 0.08) |  |
| Georgia | 119.51 (0 to 295.43) | 2.12 (0 to 5.46) | 3.2 (0 to 8.29) | 0.09 (0 to 0.23) |  |
| Germany | 8574.19 (0 to 19483.2) | 4.12 (0 to 9.49) | 518.73 (0 to 1199.47) | 0.61 (0 to 1.4) |  |
| Ghana | 653.95 (0 to 1565.66) | 3.94 (0 to 9.28) | 24.35 (0 to 56.3) | 0.07 (0 to 0.16) |  |
| Greece | 2630.31 (0.27 to 5881.7) | 9.63 (0 to 21.53) | 166.36 (0 to 373.27) | 1.64 (0 to 3.67) |  |
| Greenland | 3.38 (0 to 7.92) | 5.11 (0 to 11.89) | 0.11 (0 to 0.27) | 0.2 (0 to 0.48) |  |
| Grenada | 8.54 (0 to 19.2) | 7.34 (0 to 16.66) | 0.31 (0 to 0.7) | 0.3 (0 to 0.68) |  |
| Guam | 22.59 (0 to 52.5) | 10.88 (0 to 24.88) | 0.76 (0 to 1.76) | 0.48 (0 to 1.1) |  |
| Guatemala | 431.62 (0 to 1038.23) | 3.69 (0 to 8.9) | 14.25 (0 to 34.16) | 0.09 (0 to 0.22) |  |
| Guinea | 171.33 (0 to 441.32) | 2.76 (0 to 6.92) | 5.3 (0 to 13.8) | 0.04 (0 to 0.1) |  |
| Guinea-Bissau | 41.16 (0 to 106.64) | 4.9 (0 to 12.4) | 1.17 (0 to 3.04) | 0.06 (0 to 0.15) |  |
| Guyana | 28.45 (0 to 68.14) | 4.13 (0 to 9.93) | 0.95 (0 to 2.33) | 0.12 (0 to 0.3) |  |
| Haiti | 271.27 (0 to 742.8) | 3.29 (0 to 8.98) | 8.19 (0 to 22.29) | 0.06 (0 to 0.17) |  |
| Honduras | 161.44 (0 to 399.19) | 2.42 (0 to 6) | 4.9 (0 to 12.17) | 0.05 (0 to 0.12) |  |
| Hungary | 471 (0 to 1087.72) | 2.59 (0 to 6.05) | 18.97 (0 to 43.9) | 0.2 (0 to 0.46) |  |
| Iceland | 23.55 (0 to 53.63) | 3.96 (0 to 9.09) | 0.98 (0 to 2.32) | 0.28 (0 to 0.66) |  |
| India | 8791.63 (0.25 to 20615.27) | 0.73 (0 to 1.73) | 276.75 (0.01 to 659.24) | 0.02 (0 to 0.05) |  |
| Indonesia | 3133.38 (0.01 to 7612.16) | 1.24 (0 to 3) | 97.25 (0 to 228.46) | 0.03 (0 to 0.08) |  |
| Iran (Islamic Republic of) | 1764.08 (0.01 to 4022.65) | 2.22 (0 to 5.18) | 64.65 (0 to 155.66) | 0.08 (0 to 0.18) |  |
| Iraq | 667.22 (0 to 1662.35) | 2.5 (0 to 6.21) | 21.56 (0 to 54.37) | 0.05 (0 to 0.13) |  |
| Ireland | 525.24 (0.04 to 1167.84) | 6.68 (0 to 14.76) | 18.54 (0 to 43.3) | 0.38 (0 to 0.88) |  |
| Israel | 475.95 (0 to 1149.04) | 3.69 (0 to 9.03) | 26.69 (0 to 65.22) | 0.28 (0 to 0.68) |  |
| Italy | 6597.86 (0.4 to 15021.42) | 3.87 (0 to 8.81) | 405.66 (0.01 to 958.69) | 0.68 (0 to 1.6) |  |
| Jamaica | 119.52 (0 to 293.98) | 3.81 (0 to 9.36) | 4.83 (0 to 11.39) | 0.17 (0 to 0.41) |  |
| Japan | 9662.57 (0.1 to 22955.8) | 2.34 (0 to 5.52) | 594.3 (0 to 1413.85) | 0.47 (0 to 1.11) |  |
| Jordan | 360.93 (0 to 846.46) | 4.71 (0 to 11.13) | 12.84 (0 to 30) | 0.1 (0 to 0.24) |  |
| Kazakhstan | 1174.5 (0.09 to 2801.8) | 6.35 (0 to 14.87) | 26.1 (0 to 59.35) | 0.14 (0 to 0.31) |  |
| Kenya | 926.06 (0.03 to 2256.44) | 4.13 (0 to 9.87) | 35.3 (0 to 85.13) | 0.07 (0 to 0.17) |  |
| Kiribati | 6.49 (0 to 16.76) | 8.75 (0 to 21.71) | 0.22 (0 to 0.56) | 0.18 (0 to 0.46) |  |
| Kuwait | 162.98 (0 to 369.53) | 4.88 (0 to 11.68) | 5.18 (0 to 12.16) | 0.11 (0 to 0.26) |  |
| Kyrgyzstan | 264.55 (0 to 641.71) | 4.93 (0 to 11.76) | 5.1 (0 to 12.43) | 0.07 (0 to 0.18) |  |
| Lao People's Democratic Republic | 232.35 (0 to 554.41) | 4.71 (0 to 11.12) | 7.71 (0 to 18.8) | 0.1 (0 to 0.25) |  |
| Latvia | 111.71 (0 to 269.22) | 2.92 (0 to 7.1) | 3.71 (0 to 9.36) | 0.2 (0 to 0.5) |  |
| Lebanon | 344.27 (0 to 814.43) | 5.58 (0 to 13.42) | 16.57 (0 to 39.62) | 0.3 (0 to 0.72) |  |
| Lesotho | 59.55 (0 to 144.53) | 5.36 (0 to 13.04) | 1.86 (0 to 4.53) | 0.1 (0 to 0.24) |  |
| Liberia | 72.31 (0 to 178.29) | 2.66 (0 to 6.39) | 2.01 (0 to 4.95) | 0.04 (0 to 0.09) |  |
| Libya | 272.28 (0 to 683.28) | 4.58 (0 to 11.37) | 9.19 (0 to 23.32) | 0.13 (0 to 0.34) |  |
| Lithuania | 179.3 (0.01 to 449.67) | 3.18 (0 to 8.17) | 4.86 (0 to 12.25) | 0.18 (0 to 0.45) |  |
| Luxembourg | 49.17 (0 to 110.59) | 4.51 (0 to 10.16) | 2.31 (0 to 5.42) | 0.36 (0 to 0.84) |  |
| Madagascar | 244.37 (0 to 606.89) | 2.32 (0 to 5.79) | 8.83 (0 to 22.49) | 0.03 (0 to 0.08) |  |
| Malawi | 151.05 (0 to 379.19) | 2.08 (0 to 5.25) | 5.76 (0 to 14.31) | 0.03 (0 to 0.07) |  |
| Malaysia | 1156.62 (0 to 2676.94) | 3.96 (0 to 9.33) | 39.45 (0 to 95.21) | 0.12 (0 to 0.3) |  |
| Maldives | 3.81 (0 to 9.45) | 1.04 (0 to 2.47) | 0.13 (0 to 0.32) | 0.03 (0 to 0.06) |  |
| Mali | 571.34 (0 to 1406.51) | 6.22 (0 to 14.95) | 18.03 (0 to 44.52) | 0.07 (0 to 0.18) |  |
| Malta | 35.56 (0 to 80.5) | 3.69 (0 to 8.4) | 1.76 (0 to 4.18) | 0.4 (0 to 0.95) |  |
| Marshall Islands | 6.1 (0 to 19.36) | 16.66 (0 to 53.06) | 0.2 (0 to 0.66) | 0.36 (0 to 1.17) |  |
| Mauritania | 166.85 (0 to 424.62) | 7.85 (0 to 19.98) | 6.39 (0 to 16.07) | 0.15 (0 to 0.37) |  |
| Mauritius | 223.17 (0 to 526.06) | 12.15 (0 to 28.37) | 9.24 (0 to 20.86) | 0.73 (0 to 1.64) |  |
| Mexico | 27742.15 (0.01 to 63301.61) | 20.96 (0 to 47.46) | 909.15 (0 to 2062.76) | 0.7 (0 to 1.6) |  |
| Micronesia (Federated States of) | 12.25 (0 to 29.53) | 16.26 (0 to 38.92) | 0.42 (0 to 1.04) | 0.41 (0 to 1.01) |  |
| Monaco | 7.61 (0 to 16.98) | 7.47 (0 to 16.63) | 0.39 (0 to 0.9) | 1.04 (0 to 2.37) |  |
| Mongolia | 370.45 (0.02 to 881.97) | 13.98 (0 to 32.11) | 7.45 (0 to 16.92) | 0.22 (0 to 0.51) |  |
| Montenegro | 57.19 (0 to 137.17) | 6.36 (0 to 15.37) | 2.15 (0 to 5.17) | 0.35 (0 to 0.84) |  |
| Morocco | 1648.03 (0 to 4101.28) | 4.68 (0 to 11.59) | 60.62 (0 to 148.18) | 0.16 (0 to 0.4) |  |
| Mozambique | 145.73 (0 to 372.56) | 1.38 (0 to 3.51) | 5.41 (0 to 13.85) | 0.02 (0 to 0.04) |  |
| Myanmar | 1273.23 (0 to 2887.5) | 2.5 (0 to 5.76) | 40.84 (0 to 94.34) | 0.07 (0 to 0.17) |  |
| Namibia | 97.19 (0 to 238.29) | 6.43 (0 to 15.56) | 2.68 (0 to 6.84) | 0.11 (0 to 0.28) |  |
| Nauru | 1.15 (0 to 2.82) | 19.18 (0 to 45.94) | 0.04 (0 to 0.1) | 0.36 (0 to 0.87) |  |
| Nepal | 796.43 (0 to 1914.83) | 3.31 (0 to 7.9) | 22.54 (0 to 54.09) | 0.07 (0 to 0.17) |  |
| Netherlands | 1492.96 (0.06 to 3418.66) | 4.17 (0 to 9.51) | 76 (0 to 183.01) | 0.44 (0 to 1.06) |  |
| New Zealand | 599.27 (0.02 to 1346.82) | 7.29 (0 to 16.56) | 26.5 (0 to 58.25) | 0.51 (0 to 1.13) |  |
| Nicaragua | 203.58 (0 to 471.06) | 3.85 (0 to 8.9) | 6.48 (0 to 15.32) | 0.1 (0 to 0.23) |  |
| Niger | 457.03 (0 to 1195.76) | 4.82 (0 to 12.15) | 12.13 (0 to 31.52) | 0.05 (0 to 0.13) |  |
| Nigeria | 2585.99 (0.01 to 6297.82) | 2.6 (0 to 6.33) | 70.54 (0 to 178.39) | 0.03 (0 to 0.08) |  |
| Niue | 0.44 (0 to 1.16) | 20.66 (0 to 53.61) | 0.02 (0 to 0.05) | 1.15 (0 to 2.96) |  |
| North Macedonia | 72.3 (0 to 173.52) | 2.37 (0 to 5.44) | 2.13 (0 to 4.97) | 0.1 (0 to 0.23) |  |
| Northern Mariana Islands | 11.13 (0 to 26.03) | 21.48 (0 to 49.41) | 0.4 (0 to 0.96) | 0.84 (0 to 1.98) |  |
| Norway | 372.23 (0.02 to 846.13) | 3.58 (0 to 8.24) | 16.88 (0 to 38.77) | 0.31 (0 to 0.72) |  |
| Oman | 228.18 (0 to 542.18) | 9.19 (0 to 21.22) | 6.7 (0 to 15.81) | 0.14 (0 to 0.34) |  |
| Pakistan | 5959.1 (0.01 to 14776.23) | 4.15 (0 to 10.11) | 166.4 (0 to 407.03) | 0.07 (0 to 0.17) |  |
| Palau | 3.94 (0 to 9.57) | 18.14 (0 to 42.84) | 0.14 (0 to 0.35) | 0.8 (0 to 1.94) |  |
| Palestine | 66.76 (0 to 157.2) | 2.54 (0 to 6.01) | 2.36 (0 to 5.69) | 0.05 (0 to 0.11) |  |
| Panama | 378.4 (0 to 866.24) | 8.5 (0 to 19.48) | 13.51 (0 to 31.48) | 0.31 (0 to 0.73) |  |
| Papua New Guinea | 156.61 (0 to 372.93) | 2.71 (0 to 6.31) | 4.3 (0 to 9.94) | 0.04 (0 to 0.1) |  |
| Paraguay | 979.15 (0.02 to 2177.71) | 16.08 (0 to 36.27) | 34.87 (0 to 78.86) | 0.49 (0 to 1.1) |  |
| Peru | 1601.99 (0 to 3747.23) | 4.73 (0 to 11.05) | 70.54 (0 to 170.7) | 0.19 (0 to 0.47) |  |
| Philippines | 5073.72 (0.01 to 11761.86) | 5.85 (0 to 13.59) | 173.74 (0 to 406.02) | 0.15 (0 to 0.36) |  |
| Poland | 3076.13 (0.01 to 6965.38) | 4.6 (0 to 10.29) | 100.06 (0 to 227.22) | 0.26 (0 to 0.59) |  |
| Portugal | 1707.16 (0.02 to 3836.31) | 6.61 (0 to 15.05) | 101.85 (0 to 229.2) | 0.96 (0 to 2.16) |  |
| Puerto Rico | 617.07 (0 to 1437.53) | 9.77 (0 to 22.38) | 30.37 (0 to 69.41) | 0.92 (0 to 2.11) |  |
| Qatar | 96.49 (0 to 229.76) | 10.34 (0 to 23.8) | 2.66 (0 to 6.39) | 0.09 (0 to 0.21) |  |
| Republic of Korea | 2166.99 (0 to 5080.92) | 2.34 (0 to 5.51) | 102.35 (0 to 245.81) | 0.2 (0 to 0.48) |  |
| Republic of Moldova | 95.33 (0 to 226.92) | 1.65 (0 to 4.02) | 1.16 (0 to 2.97) | 0.03 (0 to 0.08) |  |
| Romania | 2612.38 (0 to 5886.85) | 7.69 (0 to 17.46) | 81.2 (0 to 189.94) | 0.43 (0 to 1) |  |
| Russian Federation | 5634.94 (0.31 to 13584.64) | 2.41 (0 to 5.85) | 181.83 (0.01 to 441.11) | 0.13 (0 to 0.3) |  |
| Rwanda | 66.28 (0 to 159.87) | 1.15 (0 to 2.82) | 2.72 (0 to 6.67) | 0.02 (0 to 0.05) |  |
| Saint Kitts and Nevis | 7.81 (0 to 17.87) | 10.96 (0 to 24.61) | 0.29 (0 to 0.66) | 0.49 (0 to 1.13) |  |
| Saint Lucia | 25 (0 to 56.39) | 10.62 (0 to 23.8) | 0.96 (0 to 2.21) | 0.54 (0 to 1.25) |  |
| Saint Vincent and the Grenadines | 9.12 (0 to 20.61) | 6.64 (0 to 15.04) | 0.34 (0 to 0.78) | 0.3 (0 to 0.68) |  |
| Samoa | 22.49 (0 to 55.86) | 15.4 (0 to 37.79) | 0.84 (0 to 2.05) | 0.39 (0 to 0.96) |  |
| San Marino | 2.67 (0 to 6.13) | 3.37 (0 to 7.73) | 0.1 (0 to 0.25) | 0.31 (0 to 0.75) |  |
| Sao Tome and Principe | 1.93 (0 to 4.63) | 1.8 (0 to 4.26) | 0.08 (0 to 0.18) | 0.04 (0 to 0.09) |  |
| Saudi Arabia | 2528.61 (0 to 6041.95) | 9.37 (0 to 22.72) | 70.23 (0 to 167.01) | 0.19 (0 to 0.44) |  |
| Senegal | 344.98 (0 to 854.53) | 4.14 (0 to 9.98) | 11.77 (0 to 28.5) | 0.07 (0 to 0.18) |  |
| Serbia | 391.06 (0 to 934.22) | 2.48 (0 to 5.93) | 17.28 (0 to 40.59) | 0.19 (0 to 0.46) |  |
| Seychelles | 5.01 (0 to 11.69) | 4.19 (0 to 9.75) | 0.19 (0 to 0.44) | 0.18 (0 to 0.42) |  |
| Sierra Leone | 47 (0 to 116.64) | 1.18 (0 to 2.9) | 1.5 (0 to 3.7) | 0.02 (0 to 0.04) |  |
| Singapore | 322.23 (0 to 735.66) | 3.8 (0 to 8.69) | 14.74 (0 to 34.37) | 0.26 (0 to 0.6) |  |
| Slovakia | 397.01 (0 to 903.59) | 4.32 (0 to 9.9) | 13.82 (0 to 32.43) | 0.25 (0 to 0.6) |  |
| Slovenia | 157.05 (0 to 361.2) | 3.56 (0 to 8.26) | 6.42 (0 to 15.54) | 0.31 (0 to 0.75) |  |
| Solomon Islands | 13.53 (0 to 32.39) | 3.44 (0 to 7.9) | 0.39 (0 to 0.91) | 0.06 (0 to 0.13) |  |
| Somalia | 329.05 (0 to 839.7) | 5.51 (0 to 14.23) | 11.44 (0 to 29.52) | 0.05 (0 to 0.14) |  |
| South Africa | 3876.82 (0.04 to 8704.17) | 7.83 (0 to 17.32) | 113.19 (0 to 251.7) | 0.2 (0 to 0.44) |  |
| South Sudan | 176.62 (0 to 455.8) | 4.73 (0 to 11.73) | 6.75 (0 to 17) | 0.07 (0 to 0.18) |  |
| Spain | 3699.03 (0 to 8557.66) | 3.42 (0 to 8.07) | 214.47 (0 to 512.5) | 0.47 (0 to 1.13) |  |
| Sri Lanka | 133.59 (0 to 317.47) | 0.5 (0 to 1.2) | 4.97 (0 to 12.39) | 0.02 (0 to 0.06) |  |
| Sudan | 859.07 (0 to 2198.28) | 3.64 (0 to 9.07) | 25 (0 to 61.57) | 0.06 (0 to 0.14) |  |
| Suriname | 33.34 (0 to 78.74) | 5.2 (0 to 12.19) | 1.13 (0 to 2.69) | 0.19 (0 to 0.46) |  |
| Sweden | 751.56 (0.04 to 1801.99) | 3.2 (0 to 7.62) | 42.8 (0 to 106.01) | 0.41 (0 to 1.02) |  |
| Switzerland | 740.41 (0.02 to 1693.76) | 3.75 (0 to 8.55) | 39.99 (0 to 93.2) | 0.45 (0 to 1.04) |  |
| Syrian Arab Republic | 662.6 (0 to 1608.98) | 4.99 (0 to 12.02) | 23.68 (0 to 58.58) | 0.17 (0 to 0.42) |  |
| Taiwan (Province of China) | 5018.3 (0 to 11316.11) | 11.69 (0 to 26.16) | 227.07 (0 to 497.97) | 0.96 (0 to 2.11) |  |
| Tajikistan | 45.38 (0 to 114.4) | 0.7 (0 to 1.71) | 0.35 (0 to 0.86) | 0 (0 to 0.01) |  |
| Thailand | 3599.56 (0 to 8907.98) | 3.3 (0 to 8.15) | 141.71 (0 to 343.02) | 0.21 (0 to 0.51) |  |
| Timor-Leste | 16.93 (0 to 42.4) | 2 (0 to 5.01) | 0.62 (0 to 1.56) | 0.04 (0 to 0.11) |  |
| Togo | 70.03 (0 to 171.9) | 1.94 (0 to 4.63) | 2.37 (0 to 5.85) | 0.03 (0 to 0.07) |  |
| Tokelau | 0.16 (0 to 0.38) | 10.81 (0 to 25.97) | 0.01 (0 to 0.02) | 0.51 (0 to 1.29) |  |
| Tonga | 5.14 (0 to 13.13) | 6.37 (0 to 16.11) | 0.19 (0 to 0.46) | 0.18 (0 to 0.44) |  |
| Trinidad and Tobago | 128.75 (0 to 295.14) | 6.76 (0 to 15.5) | 4.84 (0 to 11.46) | 0.35 (0 to 0.82) |  |
| Tunisia | 383.68 (0 to 935.98) | 2.94 (0 to 7.15) | 15.67 (0 to 37.41) | 0.13 (0 to 0.32) |  |
| Turkey | 3485.38 (0 to 8634.68) | 3.77 (0 to 9.31) | 145.95 (0 to 353.77) | 0.17 (0 to 0.42) |  |
| Turkmenistan | 561.53 (0.13 to 1365.96) | 12.4 (0 to 30.01) | 12.67 (0 to 31.12) | 0.25 (0 to 0.6) |  |
| Tuvalu | 1.23 (0 to 2.94) | 11.69 (0 to 29.04) | 0.05 (0 to 0.12) | 0.37 (0 to 0.93) |  |
| Uganda | 374.93 (0 to 944.18) | 2.62 (0 to 6.43) | 14.57 (0 to 35.87) | 0.03 (0 to 0.08) |  |
| Ukraine | 1112.36 (0.03 to 2740.55) | 1.54 (0 to 3.78) | 11.16 (0 to 29.72) | 0.03 (0 to 0.07) |  |
| United Arab Emirates | 383.28 (0 to 929.28) | 8.6 (0 to 20.2) | 8.14 (0 to 19.52) | 0.08 (0 to 0.2) |  |
| United Kingdom | 2324.1 (0.13 to 5349.65) | 1.85 (0 to 4.31) | 82.58 (0 to 201.25) | 0.12 (0 to 0.3) |  |
| United Republic of Tanzania | 732.07 (0 to 1740.64) | 2.78 (0 to 6.48) | 26.37 (0 to 61.8) | 0.05 (0 to 0.11) |  |
| **United States of America** | **68860.04 (4.56 to 146880.49)** | **12.85 (0 to 27.26)** | **2967.13 (0.11 to 6621.52)** | **0.89 (0 to 1.99)** |  |
| United States Virgin Islands | 8.95 (0 to 21.09) | 6.12 (0 to 14.26) | 0.35 (0 to 0.84) | 0.41 (0 to 0.98) |  |
| Uruguay | 411.21 (0.02 to 964.25) | 7.17 (0 to 16.63) | 23.24 (0 to 55.43) | 0.68 (0 to 1.63) |  |
| Uzbekistan | 1675.63 (0.03 to 4169.07) | 5.55 (0 to 13.57) | 32.9 (0 to 78.99) | 0.1 (0 to 0.23) |  |
| Vanuatu | 25.82 (0 to 60.51) | 13.6 (0 to 31.82) | 0.79 (0 to 1.91) | 0.25 (0 to 0.61) |  |
| Venezuela (Bolivarian Republic of) | 2705.23 (0 to 6421.36) | 8.83 (0 to 21.01) | 95.58 (0 to 229.78) | 0.36 (0 to 0.86) |  |
| Viet Nam | 4190.61 (0 to 9556.82) | 4.22 (0 to 9.64) | 164.49 (0 to 383.24) | 0.16 (0 to 0.38) |  |
| Yemen | 266.59 (0 to 663.06) | 1.79 (0 to 4.5) | 8.45 (0 to 22.29) | 0.03 (0 to 0.07) |  |
| Zambia | 314.15 (0 to 809.78) | 4.27 (0 to 10.45) | 10.86 (0 to 26.59) | 0.06 (0 to 0.14) |  |
| Zimbabwe | 359.61 (0 to 862) | 4.37 (0 to 10.18) | 9.44 (0 to 22.37) | 0.06 (0 to 0.14) |  |

Abbreviations: ASR: age-standardized rates; DALYs: Disability Adjusted Life Years; CKD: chronic kidney disease; ASDR: age-standardized DALYs rate; ASMR: age-standardized mortality rate.

**Supplementary Table 4:** Changes in DALYs of T2DM related to CKD due to high red meat intake according to disease categories and population-level determinants from 1990 to 2021 globally.

| **Disease** | **Location** | **Overll difference** | **Aging (%)** | **Population (%)** | **Epidemiological change (%)** |
| --- | --- | --- | --- | --- | --- |
| CKD due to T2DM-Both | Global | 229648.71 | 20.47 | 57.21 | 22.32 |
|  | **High SDI** | **55827.12** | **30.17** | **40.61** | **29.22** |
|  | High-middle SDI | 46468.19 | 31.34 | 51.87 | 16.79 |
|  | Middle SDI | 102119.56 | 24 | 51.29 | 24.72 |
|  | Low-middle SDI | 20968.23 | 10.63 | 65.25 | 24.12 |
|  | Low-SDI | 4164.08 | -8.27 | 142.91 | -34.64 |
| CKD due to T2DM-Male | Global | 120756.03 | 21.76 | 55.27 | 22.98 |
|  | High SDI | 30228.21 | 33.08 | 40.25 | 26.67 |
|  | High-middle SDI | 22607.6 | 36.43 | 53.73 | 9.83 |
|  | Middle SDI | 55099.32 | 23.05 | 48.22 | 28.73 |
|  | Low-middle SDI | 10777.16 | 8.44 | 66.04 | 25.51 |
|  | Low-SDI | 1993.69 | -14.47 | 161.81 | -47.34 |
| CKD due to T2DM-Female | Global | 108892.68 | 19.69 | 59.33 | 20.98 |
|  | High SDI | 25598.91 | 29.62 | 41.19 | 29.19 |
|  | High-middle SDI | 23860.59 | 28.22 | 50.23 | 21.54 |
|  | Middle SDI | 47020.24 | 25.06 | 54.73 | 20.21 |
|  | Low-middle SDI | 10191.07 | 12.54 | 64.01 | 23.45 |
|  | Low-SDI | 2170.39 | -3.38 | 124.87 | -21.49 |

Abbreviations: DALYs: Disability Adjusted Life Years; CKD: chronic kidney disease; T2DM: type 2 diabetes mellitus; SDI: sociodemographic index.

**Supplementary Table 5:** Changes in Deaths of T2DM related to CKD due to high red meat intake according to disease categories and population-level determinants from 1990 to 2021 globally.

| **Disease** | **Location** | **Overll difference** | **Aging (%)** | **Population (%)** | **Epidemiological change (%)** |
| --- | --- | --- | --- | --- | --- |
| CKD due to T2DM-Both | Global | 9849.38 | 24.78 | 48.34 | 26.88 |
|  | **High SDI** | **2927.47** | **34.57** | **30.57** | **34.86** |
|  | High-middle SDI | 2006.86 | 36.20 | 42.22 | 21.58 |
|  | Middle SDI | 4053.51 | 28.61 | 46.18 | 25.21 |
|  | Low-middle SDI | 722.2 | 15.06 | 63.33 | 21.62 |
|  | Low-SDI | 134.75 | -7.04 | 147.61 | -40.57 |
| CKD due to T2DM-Male | Global | 4990.64 | 26.72 | 46.92 | 26.36 |
|  | High SDI | 1482.83 | 37.33 | 30.41 | 32.26 |
|  | High-middle SDI | 934.43 | 43.63 | 44.96 | 11.41 |
|  | Middle SDI | 2151.34 | 27.65 | 43.14 | 29.21 |
|  | Low-middle SDI | 359.16 | 12.21 | 65.30 | 22.49 |
|  | Low-SDI | 60.76 | -15.32 | 178.70 | -63.38 |
| CKD due to T2DM-Female | Global | 4858.74 | 23.81 | 49.80 | 26.39 |
|  | High SDI | 1444.65 | 34.83 | 30.60 | 34.57 |
|  | High-middle SDI | 1072.43 | 32.34 | 39.98 | 27.68 |
|  | Middle SDI | 1902.17 | 29.67 | 49.57 | 20.76 |
|  | Low-middle SDI | 363.03 | 17.30 | 61.04 | 21.66 |
|  | Low-SDI | 73.99 | -1.23 | 121.31 | -20.09 |

Abbreviations: CKD: chronic kidney disease; T2DM: type 2 diabetes mellitus; SDI: sociodemographic index.

**Supplementary Table 6:** The cases, ASDR and EAPC of CKD DALYs from 1990 to 2021 in China.

| **DALYs** | **1990** | | **2021** | | **EAPC in ASDR** **(95% CI)** |
| --- | --- | --- | --- | --- | --- |
|  | **Cases (95% UI)** | **ASDR/100000(95% UI)** | **Cases (95% UI)** | **ASDR/100000(95% UI)** |  |
| China | 40019.76 (0.19 to 89533.17) | 4.95 (0 to 11.26) | 141230.18 (4.21 to 302598.07) | 6.76 (0 to 14.42) | 1.31 (1.13 to 1.49) |
| **Sex** |  |  |  |  |  |
| Male | 19735.5 (0.03 to 47481.17) | 5.15 (0 to 12.14) | 72577.96 (0.74 to 158817.62) | 6.23 (0 to 14.23) | 1.57 (1.37 to 1.77) |
| Female | 20284.26 (0.03 to 46116.83) | 4.87 (0 to 11.13) | 68652.22 (1.5 to 157724.08) | 7.49 (0 to 16.59) | 1.03 (0.86 to 1.2) |
| **Cause** |  |  |  |  |  |
| Diabetes mellitus type 2 | 39430.99 (0 to 88050.62) | 4.85 (0 to 10.91) | 137647.46 (0 to 298465.74) | 6.56 (0 to 14.28) | 1.26 (1.08 to 1.44) |
| Hypertension | 331.55 (0.02 to 2813.49) | 0.06 (0 to 0.35) | 2131.99 (0.84 to 12925.64) | 0.12 (0 to 0.64) | 3.06 (2.79 to 3.32) |
| **Glomerulonephritis** | **73.43 (0 to 507.61)** | **0.01 (0 to 0.06)** | **357.66 (0.14 to 2238.6)** | **0.02 (0 to 0.12)** | **3.82 (3.53 to 4.1)** |
| Other and unspecified causes | 183.8 (0.01 to 1450.66) | 0.04 (0 to 0.21) | 1093.07 (0.63 to 6541.02) | 0.07 (0 to 0.37) | 2.79 (2.48 to 3.1) |
| **Age** |  |  |  |  |  |
| 25-29 | 434.14 (0 to 1564.09) | 0.4 (0 to 1.42) | 423.87 (0.19 to 1532.39) | 0.49 (0 to 1.77) | 0.97 (0.82 to 1.12) |
| 30-34 | 755.94 (0 to 2307.58) | 0.86 (0 to 2.61) | 1238.9 (0 to 3838.55) | 1.02 (0 to 3.17) | 0.75 (0.62 to 0.87) |
| 35-39 | 1863.55 (0 to 5154.31) | 2.04 (0 to 5.64) | 2426.12 (0 to 6534.58) | 2.29 (0 to 6.17) | 0.28 (0.12 to 0.44) |
| 40-44 | 2386.21 (0 to 6972.49) | 3.56 (0 to 10.39) | 3789.21 (0 to 10079.38) | 4.14 (0 to 11.01) | 0.58 (0.43 to 0.72) |
| 45-49 | 2658.44 (0 to 7236.69) | 5.15 (0 to 14.02) | 6752.64 (0 to 17544.87) | 6.12 (0 to 15.9) | 0.94 (0.75 to 1.13) |
| 50-54 | 4018.96 (0 to 10232.9) | 8.42 (0 to 21.45) | 12262.9 (0 to 29704.92) | 10.15 (0 to 24.58) | 0.81 (0.67 to 0.95) |
| 55-59 | 5247.2 (0 to 12757.17) | 12.1 (0 to 29.42) | 17185.09 (0 to 41954.76) | 15.63 (0 to 38.16) | 1.06 (0.89 to 1.23) |
| 60-64 | 5434.6 (0 to 13470.42) | 15.38 (0 to 38.12) | 15794.65 (0 to 37813.04) | 21.63 (0 to 51.8) | 1.59 (1.36 to 1.82) |
| 65-69 | 5580.46 (0 to 13904.33) | 20.45 (0 to 50.97) | 22683 (0 to 52843.07) | 29.57 (0 to 68.89) | 1.65 (1.41 to 1.9) |
| 70-74 | 5053.69 (0 to 13249.68) | 26.86 (0 to 70.41) | 21690.71 (0 to 54467.68) | 40.7 (0 to 102.2) | 1.77 (1.52 to 2.02) |
| 75-79 | 3687.33 (0 to 9279.18) | 32.4 (0 to 81.53) | 16356.63 (0 to 41206.55) | 49.39 (0 to 124.42) | 1.82 (1.56 to 2.08) |
| 80-84 | 1674.08 (0 to 4197.33) | 31.6 (0 to 79.24) | 10159.03 (0 to 24810.22) | 51.33 (0 to 125.36) | 1.97 (1.69 to 2.25) |
| 85-89 | 914.13 (0 to 2285.73) | 54.19 (0 to 135.5) | 6690.6 (0 to 17421.92) | 70.24 (0 to 182.89) | 0.8 (0.64 to 0.96) |
| 90-94 | 268.08 (0.01 to 748.57) | 87.37 (0 to 243.97) | 3004.77 (0.05 to 7617.97) | 102.48 (0 to 259.82) | 0.37 (0.27 to 0.48) |
| 95 plus | 42.94 (0 to 118.19) | 106.04 (0.01 to 291.88) | 772.05 (0.05 to 2045.78) | 120.8 (0.01 to 320.1) | 0.29 (0.17 to 0.41) |

Abbreviations: ASDR: age-standardised DALYs rate; DALYs: Disability Adjusted Life Years; EAPC: estimated annual percentage change; CKD: chronic kidney disease; UI: uncertainty interval; CI: confidence interval.

**Supplementary Table 7:** The cases, ASMR and EAPC of CKD Deaths from 1990 to 2021 in China.

| **Deaths** | **1990** | | **2021** | | **EAPC in ASMR** **(95% CI)** |
| --- | --- | --- | --- | --- | --- |
|  | **Cases (95% UI)** | **ASMR/100000(95% UI)** | **Cases (95% UI)** | **ASMR/100000(95% UI)** |  |
| China | 1316.97 (0.01 to 2985.29) | 0.21 (0 to 0.48) | 5365.8 (0.07 to 11824.07) | 0.28 (0 to 0.61) | 1.12 (0.96 to 1.27) |
| **Sex** |  |  |  |  |  |
| Male | 625.61 (0 to 1522.03) | 0.23 (0 to 0.56) | 2755.72 (0.02 to 6344.91) | 0.33 (0 to 0.78) | 1.47 (1.28 to 1.67) |
| Female | 691.36 (0 to 1554.82) | 0.2 (0 to 0.45) | 2610.08 (0.03 to 6132.43) | 0.24 (0 to 0.57) | 0.75 (0.61 to 0.89) |
| **Cause** |  |  |  |  |  |
| Diabetes mellitus type 2 | 1301.2 (0 to 2940.78) | 0.2 (0 to 0.46) | 5253.06 (0 to 11754.16) | 0.27 (0 to 0.61) | 1.09 (0.93 to 1.25) |
| Hypertension | 13.36 (0 to 93.07) | 0 (0 to 0.02) | 98.89 (0.04 to 487.92) | 0.01 (0 to 0.03) | 2.24 (2 to 2.48) |
| **Glomerulonephritis** | **1.23 (0 to 8.82)** | **0 (0 to 0)** | **6.63 (0 to 39.77)** | **0 (0 to 0)** | **2.74 (2.49 to 2.99)** |
| Other and unspecified causes | 1.19 (0 to 7.99) | 0 (0 to 0) | 7.22 (0 to 40.59) | 0 (0 to 0) | 2.32 (2.09 to 2.55) |
| **Age** |  |  |  |  |  |
| 25-29 | 4.59 (0 to 17.24) | 0 (0 to 0.02) | 4.59 (0 to 16.09) | 0.01 (0 to 0.02) | 0.88 (0.72 to 1.05) |
| 30-34 | 10.38 (0 to 34.15) | 0.01 (0 to 0.04) | 16.47 (0 to 50.87) | 0.01 (0 to 0.04) | 0.52 (0.35 to 0.69) |
| 35-39 | 29.68 (0 to 85.56) | 0.03 (0 to 0.09) | 36.36 (0 to 99.66) | 0.03 (0 to 0.09) | -0.05 (-0.26 to 0.17) |
| 40-44 | 42.14 (0 to 123.28) | 0.06 (0 to 0.18) | 61.47 (0 to 166.86) | 0.07 (0 to 0.18) | 0.23 (0.06 to 0.4) |
| 45-49 | 50.27 (0 to 134.85) | 0.1 (0 to 0.26) | 113.13 (0 to 291.29) | 0.1 (0 to 0.26) | 0.55 (0.33 to 0.76) |
| 50-54 | 86.22 (0 to 221.91) | 0.18 (0 to 0.47) | 229.84 (0 to 585.09) | 0.19 (0 to 0.48) | 0.29 (0.16 to 0.42) |
| 55-59 | 126.64 (0 to 312.62) | 0.29 (0 to 0.72) | 369.97 (0 to 946.65) | 0.34 (0 to 0.86) | 0.58 (0.44 to 0.72) |
| 60-64 | 151.06 (0 to 385.81) | 0.43 (0 to 1.09) | 410.24 (0 to 985.6) | 0.56 (0 to 1.35) | 1.29 (1.09 to 1.49) |
| 65-69 | 187.57 (0 to 465.06) | 0.69 (0 to 1.7) | 732.01 (0 to 1712.62) | 0.95 (0 to 2.23) | 1.41 (1.2 to 1.62) |
| 70-74 | 210.56 (0 to 554.41) | 1.12 (0 to 2.95) | 882.11 (0 to 2244.47) | 1.66 (0 to 4.21) | 1.59 (1.37 to 1.81) |
| 75-79 | 193.8 (0 to 481.06) | 1.7 (0 to 4.23) | 843.67 (0 to 2151.13) | 2.55 (0 to 6.5) | 1.68 (1.44 to 1.92) |
| 80-84 | 111.99 (0 to 279.87) | 2.11 (0 to 5.28) | 684.03 (0 to 1693.39) | 3.46 (0 to 8.56) | 1.91 (1.64 to 2.18) |
| 85-89 | 80.15 (0 to 199.73) | 4.75 (0 to 11.84) | 592.85 (0 to 1592.6) | 6.22 (0 to 16.72) | 0.74 (0.59 to 0.9) |
| 90-94 | 27.29 (0 to 72.8) | 8.89 (0 to 23.73) | 304.56 (0 to 752.24) | 10.39 (0 to 25.66) | 0.28 (0.17 to 0.39) |
| 95 plus | 4.64 (0 to 12.03) | 11.46 (0 to 29.7) | 84.5 (0 to 209.95) | 13.22 (0 to 32.85) | 0.25 (0.12 to 0.38) |

Abbreviations: ASMR: age standardised mortality rate; EAPC: estimated annual percentage change; CKD: chronic kidney disease; UI: uncertainty interval; CI: confidence interval.

**Supplementary Table 8:** The cases, ASDR and EAPC of CKD DALYs from 1990 to 2021 in the United States of America.

| **DALYs** | **1990** | | **2021** | | **EAPC in ASDR** **(95% CI)** |
| --- | --- | --- | --- | --- | --- |
|  | **Cases (95% UI)** | **ASDR/100000(95% UI)** | **Cases (95% UI)** | **ASDR/100000(95% UI)** |  |
| United States of America | 20172.98 (3.49 to 44354.61) | 6.55 (0 to 14.44) | 68860.04 (4.56 to 146880.49) | 12.85 (0 to 27.26) | 2.46 (2.27 to 2.65) |
| **Sex** |  |  |  |  |  |
| Male | 10388.06 (2.04 to 22992.18) | 7.92 (0 to 17.55) | 38764.78 (1.81 to 82224.4) | 15.73 (0 to 33.33) | 2.57 (2.32 to 2.81) |
| Female | 9784.92 (1.46 to 21862.34) | 5.58 (0 to 12.45) | 30095.26 (2.7 to 66289.85) | 10.49 (0 to 22.62) | 2.23 (1.98 to 2.47) |
| **Cause** |  |  |  |  |  |
| Diabetes mellitus type 2 | 16714.19 (0 to 35325.49) | 5.45 (0 to 11.42) | 52450.85 (0 to 108060.82) | 9.62 (0 to 19.57) | 2.09 (1.87 to 2.32) |
| Hypertension | 1487.46 (1.21 to 5082.42) | 0.46 (0 to 1.6) | 8995.76 (2.19 to 25747.64) | 1.64 (0 to 4.61) | 4.44 (4.2 to 4.68) |
| **Glomerulonephritis** | **755.84 (0.97 to 2604.05)** | **0.25 (0 to 0.87)** | **3892.08 (1.56 to 11211.33)** | **0.89 (0 to 2.51)** | **4.67 (4.39 to 4.95)** |
| Other and unspecified causes | 1215.49 (1.15 to 4708.91) | 0.39 (0 to 1.47) | 3521.36 (0.97 to 10097.38) | 0.71 (0 to 2.07) | 2.03 (1.53 to 2.54) |
| **Age** |  |  |  |  |  |
| 25-29 | 353.14 (0.05 to 953.21) | 1.62 (0 to 4.39) | 739.28 (0 to 1823.8) | 3.28 (0 to 8.1) | 2.93 (2.64 to 3.23) |
| 30-34 | 590.04 (0.11 to 1499.28) | 2.64 (0 to 6.71) | 1124.92 (0.04 to 2824.88) | 4.89 (0 to 12.29) | 3.18 (2.82 to 3.53) |
| 35-39 | 907.73 (0.5 to 2230) | 4.45 (0 to 10.94) | 1703 (0.28 to 4211.24) | 7.71 (0 to 19.08) | 3.25 (2.85 to 3.66) |
| 40-44 | 1123.62 (0.16 to 2826.37) | 6.22 (0 to 15.64) | 2553.04 (0.17 to 5867.32) | 12.15 (0 to 27.93) | 3.26 (2.94 to 3.58) |
| 45-49 | 1175.49 (0.01 to 2846.14) | 8.39 (0 to 20.33) | 3549.66 (0.42 to 8213.93) | 18.03 (0 to 41.71) | 3.49 (3.19 to 3.79) |
| 50-54 | 1367.32 (0.08 to 2963.21) | 11.91 (0 to 25.82) | 5447.32 (0.3 to 12679.81) | 25.99 (0 to 60.49) | 3.28 (3.02 to 3.55) |
| 55-59 | 1629.67 (0.49 to 3673.77) | 15.5 (0 to 34.94) | 6679.38 (1.04 to 15493.23) | 30.77 (0 to 71.38) | 2.71 (2.45 to 2.96) |
| 60-64 | 2087.37 (0.11 to 4848.03) | 19.58 (0 to 45.48) | 7349.62 (0.96 to 17279.77) | 34.3 (0 to 80.63) | 1.81 (1.57 to 2.05) |
| 65-69 | 2403.2 (0.01 to 5850.61) | 23.84 (0 to 58.05) | 7780.73 (0.24 to 18159.35) | 41.77 (0 to 97.5) | 1.57 (1.28 to 1.86) |
| 70-74 | 2483.38 (0.11 to 6092.7) | 30.73 (0 to 75.4) | 9186.18 (0.41 to 21335.32) | 59.25 (0 to 137.61) | 1.88 (1.58 to 2.17) |
| 75-79 | 2365.65 (0.03 to 5581.2) | 38.56 (0 to 90.97) | 7642.46 (0.18 to 17684.48) | 75.32 (0 to 174.28) | 2.01 (1.76 to 2.26) |
| 80-84 | 1715.21 (0.04 to 3996.99) | 43.55 (0 to 101.49) | 5997.55 (0 to 13624.97) | 92.31 (0 to 209.71) | 2.38 (2.15 to 2.61) |
| 85-89 | 1114.14 (0.01 to 2668.87) | 54.67 (0 to 130.96) | 4470.68 (0 to 10659.03) | 117.62 (0 to 280.44) | 2.35 (2.18 to 2.53) |
| 90-94 | 539.44 (0 to 1333.76) | 70.38 (0 to 174.02) | 2896.1 (0 to 7533.07) | 155.35 (0 to 404.08) | 2.61 (2.41 to 2.81) |
| 95 plus | 317.6 (0 to 825.64) | 126.84 (0 to 329.74) | 1740.12 (0 to 4235.29) | 258.88 (0 to 630.09) | 2.35 (2.08 to 2.62) |

Abbreviations: ASDR: age-standardised DALYs rate; DALYs: Disability Adjusted Life Years; EAPC: estimated annual percentage change; CKD: chronic kidney disease; UI: uncertainty interval; CI: confidence interval.

**Supplementary Table 9:** The cases, ASMR and EAPC of CKD Deaths from 1990 to 2021 in the United States of America.

| **Deaths** | **1990** | | **2021** | | **EAPC in ASMR** **(95% CI)** |
| --- | --- | --- | --- | --- | --- |
|  | **Cases (95% UI)** | **ASMR/100000(95% UI)** | **Cases (95% UI)** | **ASMR/100000(95% UI)** |  |
| United States of America | 656.18 (0.07 to 1482.83) | 0.2 (0 to 0.45) | 2967.13 (0.11 to 6621.52) | 0.49 (0 to 1.08) | 3.11 (2.89 to 3.33) |
| **Sex** |  |  |  |  |  |
| Male | 317.4 (0.03 to 722.33) | 0.26 (0 to 0.58) | 1649.94 (0.05 to 3541.54) | 0.38 (0 to 0.85) | 3.19 (2.86 to 3.51) |
| Female | 338.78 (0.03 to 779.42) | 0.17 (0 to 0.39) | 1317.19 (0.06 to 3000.64) | 0.64 (0 to 1.36) | 2.76 (2.5 to 3.02) |
| **Cause** |  |  |  |  |  |
| Diabetes mellitus type 2 | 525.14 (0 to 1193.54) | 0.16 (0 to 0.36) | 2232.54 (0 to 4648.54) | 0.37 (0 to 0.78) | 2.91 (2.64 to 3.18) |
| **Hypertension** | **81.15 (0.03 to 262.01)** | **0.03 (0 to 0.1)** | **531.24 (0.06 to 1479.86)** | **0.16 (0 to 0.44)** | **4.24 (4.01 to 4.46)** |
| Glomerulonephritis | 23.82 (0.02 to 78.65) | 0.01 (0 to 0.02) | 129.85 (0.04 to 375.62) | 0.02 (0 to 0.07) | 4.16 (3.94 to 4.38) |
| Other and unspecified causes | 26.07 (0.01 to 89.28) | 0.01 (0 to 0.03) | 73.51 (0.01 to 202.29) | 0.01 (0 to 0.03) | 1.54 (0.8 to 2.29) |
| **Age** |  |  |  |  |  |
| 25-29 | 2.5 (0 to 6.42) | 0.01 (0 to 0.03) | 6.22 (0 to 15.42) | 0.03 (0 to 0.07) | 4.03 (3.63 to 4.45) |
| 30-34 | 5.05 (0 to 12.37) | 0.02 (0 to 0.06) | 11.26 (0 to 27.27) | 0.05 (0 to 0.12) | 4.34 (3.79 to 4.89) |
| 35-39 | 9.53 (0 to 23.48) | 0.05 (0 to 0.12) | 20.5 (0 to 49.3) | 0.09 (0 to 0.22) | 4.32 (3.68 to 4.96) |
| 40-44 | 13.1 (0 to 31.34) | 0.07 (0 to 0.17) | 37.27 (0 to 83.36) | 0.18 (0 to 0.4) | 4.43 (3.97 to 4.9) |
| 45-49 | 15.9 (0 to 38.56) | 0.11 (0 to 0.28) | 61.41 (0.01 to 143.64) | 0.31 (0 to 0.73) | 4.75 (4.32 to 5.18) |
| 50-54 | 22.31 (0 to 50.32) | 0.19 (0 to 0.44) | 111.92 (0.01 to 254.02) | 0.53 (0 to 1.21) | 4.46 (4.08 to 4.83) |
| 55-59 | 31.21 (0.01 to 71.56) | 0.3 (0 to 0.68) | 159.47 (0.02 to 366.8) | 0.73 (0 to 1.69) | 3.74 (3.42 to 4.07) |
| 60-64 | 48.74 (0 to 114.19) | 0.46 (0 to 1.07) | 210.07 (0.03 to 488.76) | 0.98 (0 to 2.28) | 2.65 (2.37 to 2.94) |
| 65-69 | 68.88 (0 to 167.2) | 0.68 (0 to 1.66) | 269.28 (0.01 to 626.75) | 1.45 (0 to 3.36) | 2.31 (1.97 to 2.64) |
| 70-74 | 88.68 (0 to 223.86) | 1.1 (0 to 2.77) | 394.71 (0.01 to 910.65) | 2.55 (0 to 5.87) | 2.57 (2.24 to 2.9) |
| 75-79 | 105.59 (0 to 256.21) | 1.72 (0 to 4.18) | 407.33 (0.01 to 948.78) | 4.01 (0 to 9.35) | 2.68 (2.41 to 2.94) |
| 80-84 | 96.14 (0 to 234.16) | 2.44 (0 to 5.95) | 408.74 (0 to 926.95) | 6.29 (0 to 14.27) | 3.09 (2.84 to 3.34) |
| 85-89 | 76.97 (0 to 178.48) | 3.78 (0 to 8.76) | 388.61 (0 to 932.21) | 10.22 (0 to 24.53) | 3.19 (2.99 to 3.4) |
| 90-94 | 43.24 (0 to 108.08) | 5.64 (0 to 14.1) | 291.5 (0 to 751.79) | 15.64 (0 to 40.33) | 3.46 (3.19 to 3.73) |
| 95 plus | 28.35 (0 to 71.26) | 11.32 (0 to 28.46) | 188.85 (0 to 465.04) | 28.09 (0 to 69.18) | 3.04 (2.71 to 3.38) |

Abbreviations: ASMR: age standardised mortality rate; EAPC: estimated annual percentage change; CKD: chronic kidney disease; UI: uncertainty interval; CI: confidence interval.

**A**


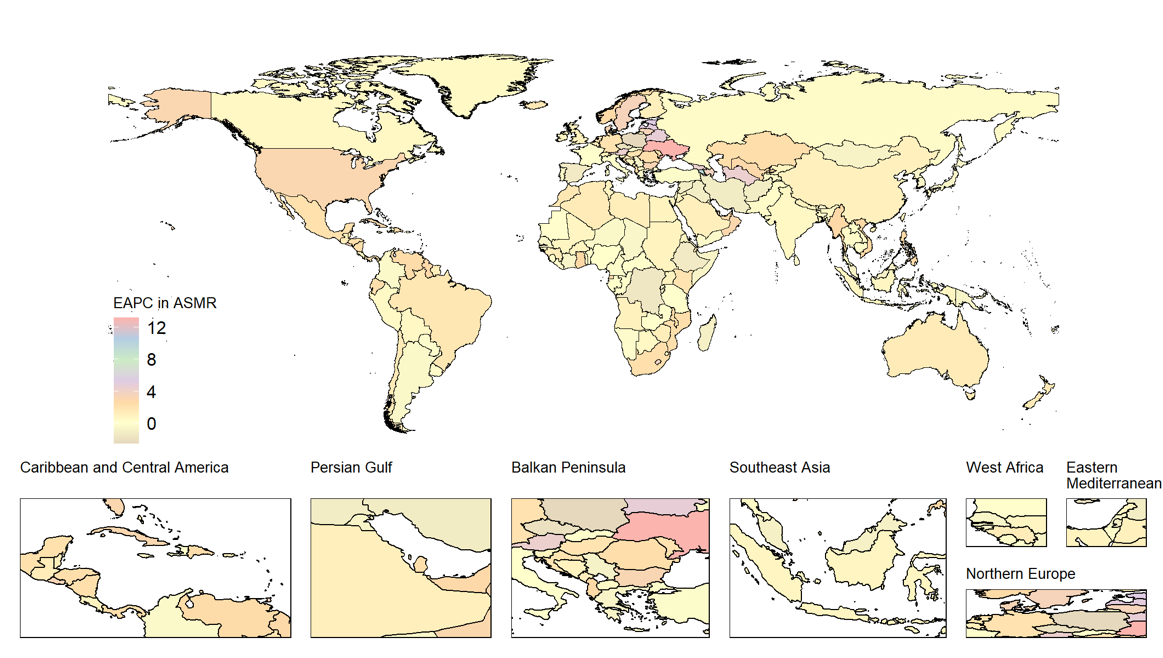


**B**


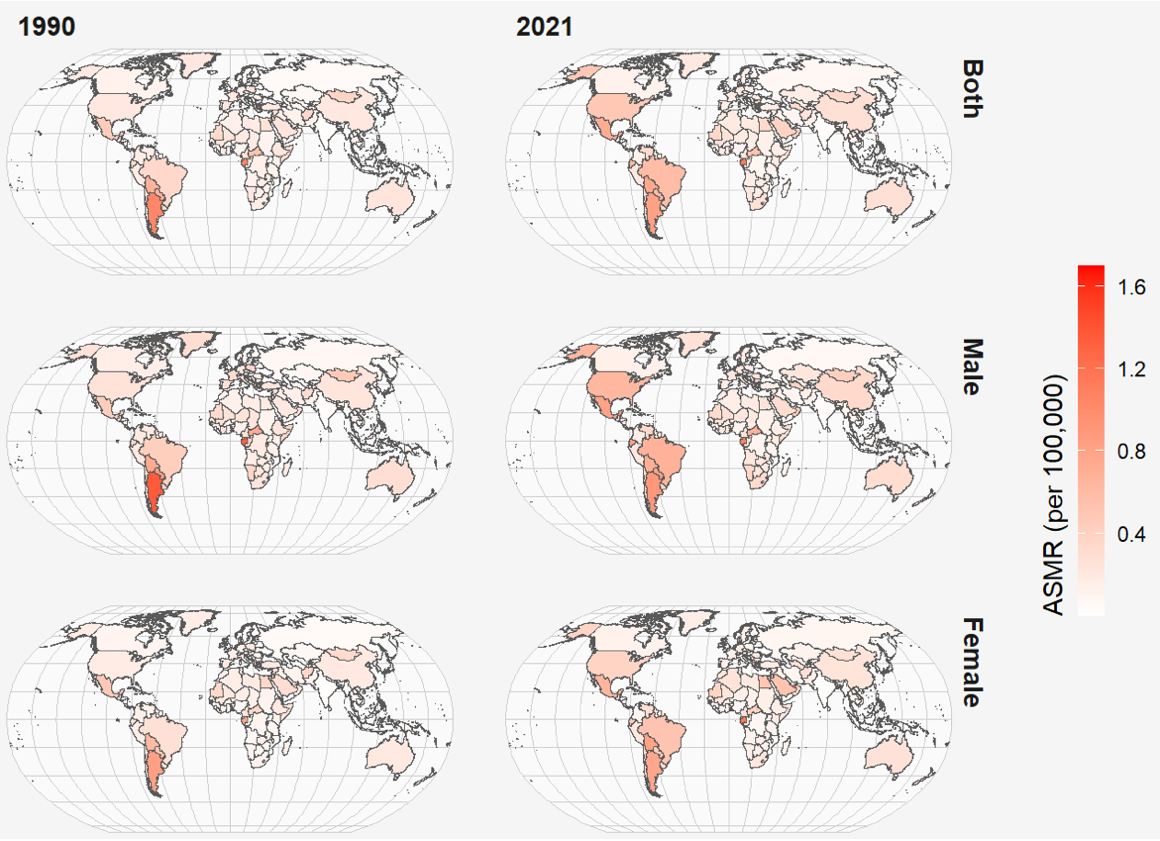


**Supplementary Figure 1:** World map of ASMR of CKD attributable to diet high in red meat across 204 countries and territories.

**(A)** The EAPC in chronic kidney disease ASMR attributable to diet high in red meat from 1990 to 2021.

**(B)** The spatial distribution of chronic kidney disease ASMR attributable to diet high in red meat by sex in 1990 and 2021.

Abbreviations: ASMR, age standardised mortality rate; CKD, chronic kidney disease; EAPC, estimated annual percentage.

**A**

**
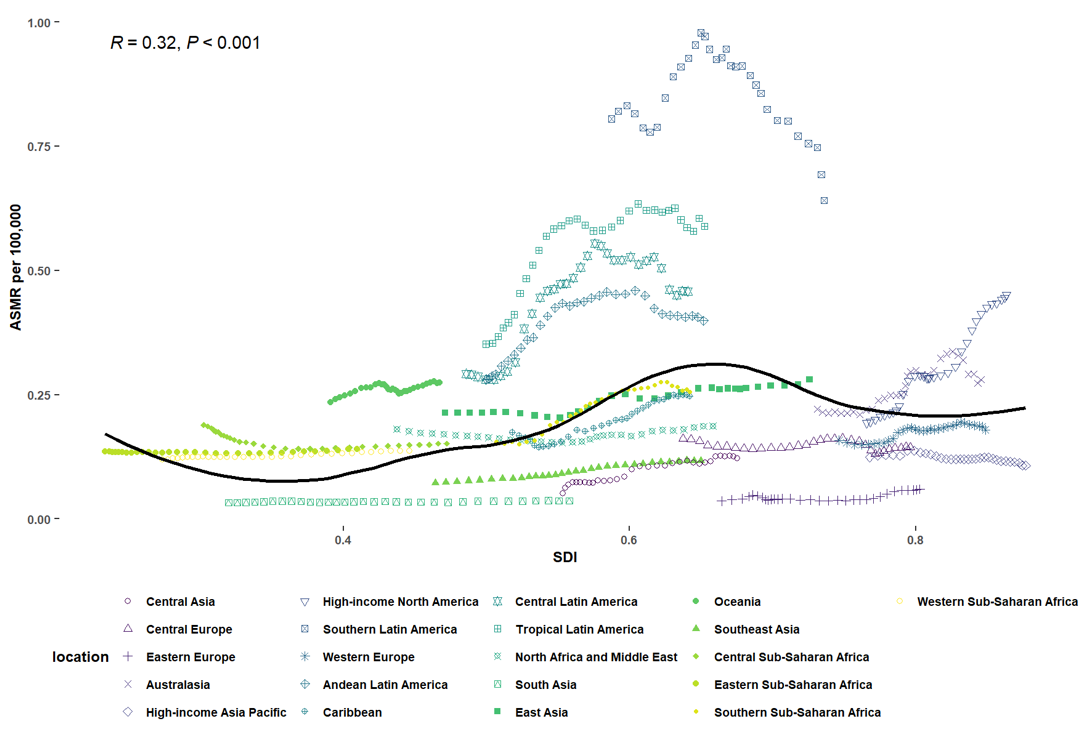
**

**B**


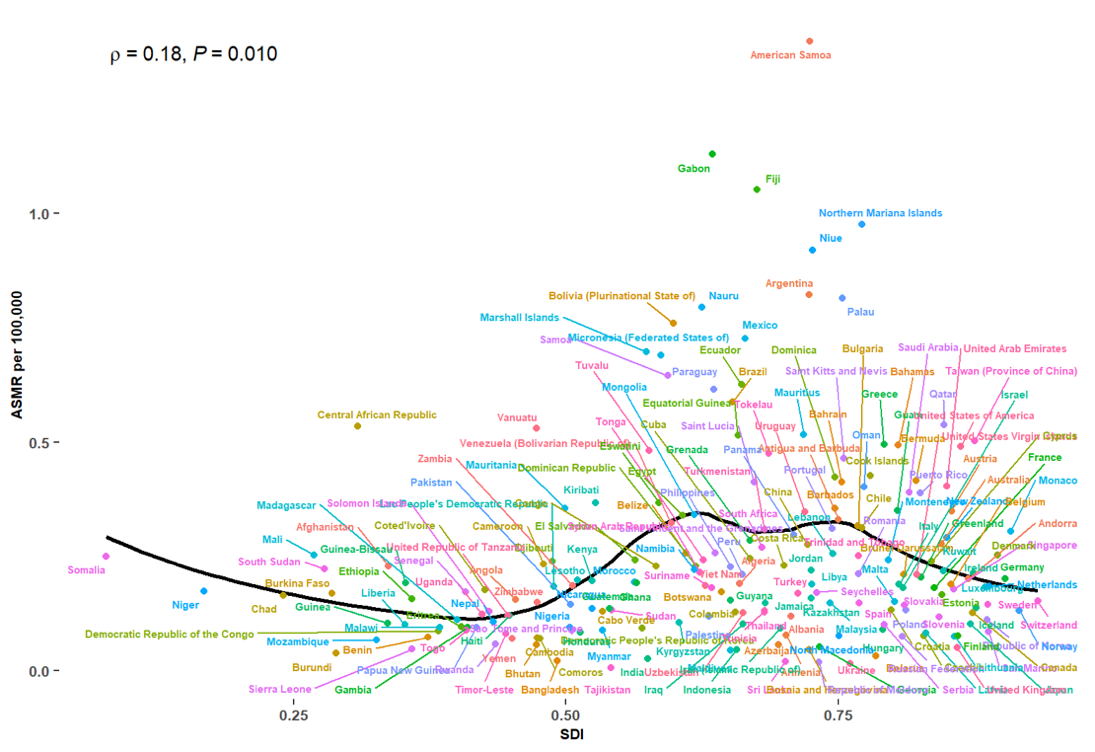


**Supplementary Figure 2:** CKD-related ASMR attributable to high red meat intake across 21 GBD regions and 204 countries, by SDI for both sexes combined, 1990–2021.

**(A)** The correlation between CKD-related ASMR attributable to high red meat intake and SDI across 21 GBD regions.

**(B)** The correlation between CKD-related ASMR attributable to high red meat intake and SDI across 204 countries.

Abbreviations: CKD, chronic kidney disease; ASMR, age standardised mortality rate; GBD, Global Burden of Disease; SDI, socio-demographic index.

**A**


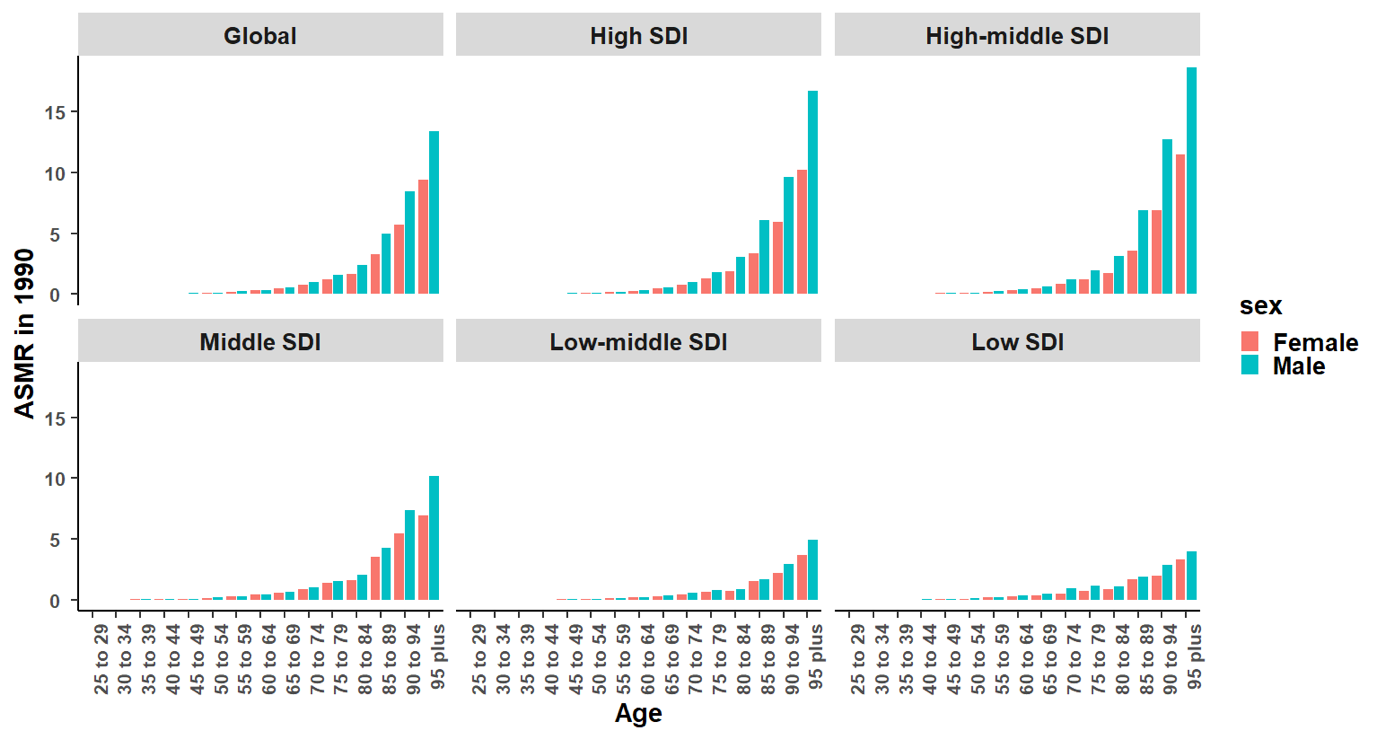


**B**


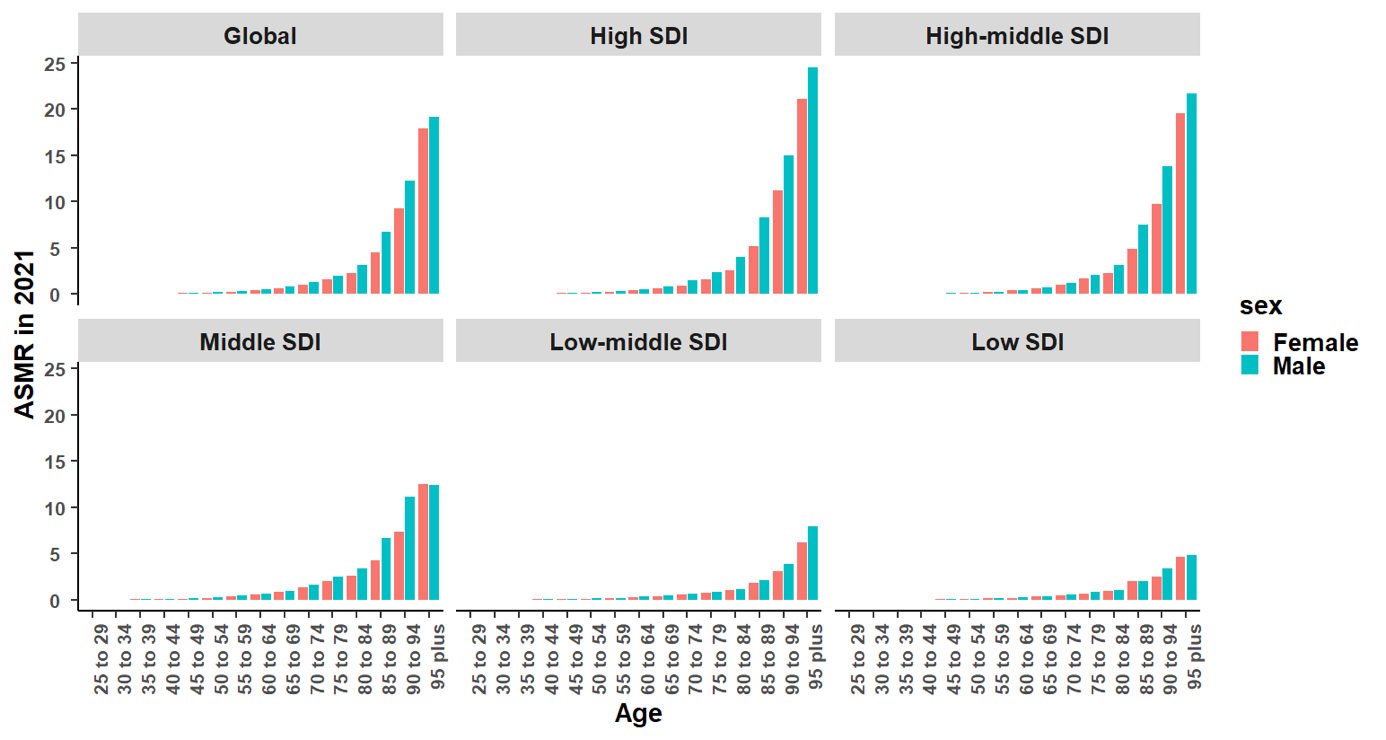


**Supplementary Figure 3:** Changes in ASMR of CKD due to high red meat intake from 1990 to 2021 by sex in different SDI quintiles.

**(A)** Changes in ASMR of CKD due to high red meat intake in 1990 by sex in different SDI quintiles.

**(B)** Changes in ASMR of CKD due to high red meat intake in 2021 by sex in different SDI quintiles.

Abbreviations: ASMR, age standardised mortality rate; CKD, Chronic Kidney Disease; SDI, sociodemographic index.
